# Supplementary material for: Protocol for the feasibility and acceptability of a brief routine weight management intervention for postnatal women embedded within the national child immunisation programme: randomised controlled cluster feasibility trial with nested qualitative study (PIMMS-WL)
Source: BMJ Open. 2020 Feb 16;10(2):e033027. doi: 10.1136/bmjopen-2019-033027 (PMC7045221; doi:10.1136/bmjopen-2019-033027)
Supplement: Supplementary data [file bmjopen-2019-033027supp001.pdf]

Table 1: Detailed study objectives

1.	In women who have recently given birth, assess the feasibility of delivering an intervention to promote self-management of weight loss, by self-monitoring of weight and signposting to an online weight management programme by practice nurses as part of the UK child immunisation programme
2.	Assess recruitment to ensure a full-scale phase III cluster trial is feasible
3.	Determine the extent of participant burden in completing the trial questionnaires
4.	Determine the potential risk for intervention contamination (whether women in the control group spontaneously access the online programme) to assess if the main trial sample size will need to be adjusted to account for this
5.	Determine levels of adherence to the intervention
6.	Collect data on immunisation uptake rates (to check there is no difference in rates in both groups, adjusted for the normal rate in the practice)
7.	To provide estimates of the variability in the primary outcome (weight) to inform the sample size for the phase III trial
8.	Using semi structured interviews explore practice nurses' views about delivering the intervention and explore any variation in intervention delivery to ascertain if any adjustments to nurse training are required
9.	Based on feedback from participants through interviews explore the acceptability of the intervention
10.	Assess the impact of the intervention on breast feeding rates and psychological health in both groups
11.	Explore the acceptability/validity of the ICECAP (ICEpop CAPability measure for Adults) for the cost effectiveness analysis in the phase III trial
