# Supplementary material for: Protocol for the feasibility and acceptability of a brief routine weight management intervention for postnatal women embedded within the national child immunisation programme: randomised controlled cluster feasibility trial with nested qualitative study (PIMMS-WL)
Source: BMJ Open. 2020 Feb 16;10(2):e033027. doi: 10.1136/bmjopen-2019-033027 (PMC7045221; doi:10.1136/bmjopen-2019-033027)
Supplement: Supplementary data [file bmjopen-2019-033027supp002.pdf]

Table 2: Inclusion and exclusion criteria

**Inclusion criteria**	**Exclusion criteria**
Aged 18 years or more	Mothers whose babies have died or have been removed from their care at birth
Women who are at least four weeks postnatal and who have not yet attended the first child immunisation appointment	Women who indicate they are already actively involved in a weight loss programme or weight management trial to lose weight
Planning to have their child immunised within the national immunisation programme	Unwilling to give consent to notify their GP
BMI 25kg/m^2 or more at the time of recruitment at the baseline home visit	Women who have been diagnosed with a serious mental health difficulty requiring hospitalisation in the past two years or been diagnosed with anorexia and/or bulimia in the past two years
Patient able and willing to provide written informed consent	
