# Supplementary material for: Protocol for the feasibility and acceptability of a brief routine weight management intervention for postnatal women embedded within the national child immunisation programme: randomised controlled cluster feasibility trial with nested qualitative study (PIMMS-WL)
Source: BMJ Open. 2020 Feb 16;10(2):e033027. doi: 10.1136/bmjopen-2019-033027 (PMC7045221; doi:10.1136/bmjopen-2019-033027)
Supplement: Supplementary data [file bmjopen-2019-033027supp003.pdf]

Appendix: Informed Consent Forms

UNIVERSITY OF BIRMINGHAM

The PIMMS-WL Trial

Feasibility and acceptability of a brief routine weight management intervention for postnatal women embedded within the national child immunisation programme in primary care: randomised controlled cluster feasibility trial with nested qualitative study.

Patient Screening Consent Form
Version 4.0 1st February 2018

PIMMS-WL Trial No:				-					Patient Initials:		

Please initial each box to indicate your consent

**1.**	I confirm that I have read and understood the information sheet for the **PIMMS-WL** trial (Version __, dated DD/MM/YYYY). I have had the opportunity to consider the information, ask questions, and have had these answered satisfactorily.	
**2.**	I understand that my participation is voluntary, that I am not obliged to take part in the full **PIMMS-WL** trial, and the standard of my medical care or legal rights will not be affected.	
**3.**	I understand that data collected that identifies me by name, including my contact details and my consent form, will be transferred from where it is collected and stored at the University of Birmingham Clinical Trials Unit (BCTU). I agree to the transfer and storage of this data for use in the **PIMMS-WL** trial.	
**4.**	I give permission for my data collected during the study to be looked at by responsible individuals from the **PIMMS-WL** trial office at the University of Birmingham Clinical Trials Unit (BCTU), from the sponsor, from regulatory authorities or from the NHS Trust, where it is relevant to my taking part in this research. I understand that all information collected will be used for this research only and that I will not be identified in any way in the analysis and reporting of the results.	
**5.**	I agree to take part in screening in order to determine my eligibility for participation for the **PIMMS-WL** study and I understand that I may not be suitable to take part as detailed in the information sheet.	

Name of Participant: ……………………………………………………………………………………….

Signature: ………………………………………………………… Date:…………………………………

Name of Person taking consent: …………………………………………………………………………

Signature: ………………………………………………………… Date:…………………………………

Original to be filed in the Investigator's Site File; 1 copy for patient;
1 copy to be kept with patient's GP record; 1 copy to be sent to BCTU
Please return a copy of this form to: The PIMMS-WL Trial, Birmingham Clinical Trials Unit (BCTU),
Institute of Applied Health Research, Public Health Building, University of Birmingham, Edgbaston, Birmingham, B15 2TT

UNIVERSITY OF BIRMINGHAM

The PIMMS-WL Trial

Feasibility and acceptability of a brief routine weight management intervention for postnatal women embedded within the national child immunisation programme in primary care: randomised controlled cluster feasibility trial with nested qualitative study.

Patient Consent Form A

Version 4.0 1st February 2018

**PIMMS-WL** Trial No:			-					Patient Initials:		

Please initial each box to indicate your consent

**1.**	I confirm that I have read and understood the information sheet for the **PIMMS-WL** trial (Version __, dated DD/MM/YYYY). I have had the opportunity to consider the information, ask questions, and have had these answered satisfactorily.	
**2.**	I understand that my participation is voluntary and that, if I take part, I may withdraw at any time, without giving any reason, and without the standard of my medical care or legal rights being affected. I understand that data collected up to my time of withdrawal may be used.	
**3.**	Data collected that identifies me and my newborn baby by name, including my contact details and a copy of my consent form, will be transferred from where it is collected and stored at the University of Birmingham Clinical Trials Unit (BCTU). I agree to the transfer and storage of this data for use in the **PIMMS-WL** trial.	
**4.**	I understand that the study researchers may contact me regarding the study.	
**5.**	I agree for the **PIMMS-WL** trial team to collect my newborn child's immunisation attendance data from my GP records for the purposes of this research study only.	
**6.**	I give permission for my data collected during the study to be looked at by responsible individuals from the **PIMMS-WL** trial office at the University of Birmingham Clinical Trials Unit (BCTU), from the sponsor, from regulatory authorities or from the NHS Trust, where it is relevant to my taking part in this research. I understand that all information collected will be used for medical research only and that I will not be identified in any way in the analysis and reporting of the results.	
**7.**	I agree to my GP being informed of my participation in the **PIMMS-WL** trial, a copy of my signed consent form to be sent, and my GP to be contacted to obtain any medical history relevant to this study.	
**8.**	I agree to take part in the **PIMMS-WL** trial.	

Name of Participant: ……………………………………………………………………………………

Signature: ………………………………………………………… Date:……………………………………

Name of Person taking consent: ……………………………………………………………………………

Signature: ………………………………………………………… Date:……………………………………

Original to be filed in the Site File; 1 copy for patient; 1 copy to be kept with patient's GP record; 1 copy to be sent to BCTU

Please return a copy of this form to: The PIMMS-WL Trial, Birmingham Clinical Trials Unit (BCTU),
Institute of Applied Health Research, Public Health Building, University of Birmingham, Edgbaston, Birmingham, B15 2TT

UNIVERSITY OF BIRMINGHAM

PIMMS-WL

The PIMMS-WL Trial

Feasibility and acceptability of a brief routine weight management intervention for postnatal women embedded within the national child immunisation programme in primary care: randomised controlled cluster feasibility trial with nested qualitative study.

Patient Consent Form B

Version 4.0 1st February 2018

PIMMS-WL Trial No: [ ][ ] - [ ][ ][ ][ ] Patient Initials: [ ][ ]

Please initial each box to indicate your consent

**1.**	I confirm that I have read and understood the information sheet for the **PIMMS-WL** trial (Version __, dated DD/MM/YYYY). I have had the opportunity to consider the information, ask questions, and have had these answered satisfactorily.	
**2.**	I understand that my participation is voluntary and that, if I take part, I may withdraw at any time, without giving any reason, and without the standard of my medical care or legal rights being affected. I understand that data collected up to my time of withdrawal may be used.	
**3.**	Data collected that identifies me and my newborn baby by name, including my contact details and a copy of my consent form, will be transferred from where it is collected and stored at the University of Birmingham Clinical Trials Unit (BCTU). I agree to the transfer and storage of this data for use in the **PIMMS-WL** trial.	
**4.**	I understand that I will be asked to complete data online using the POWeR website at the University of Southampton. I consent to the **PIMMS-WL** research team to access, transfer and store this electronic data.	
**5.**	I understand that the study researchers may contact me regarding the study.	
**6.**	I agree for the **PIMMS-WL** trial team to collect my newborn child's immunisation attendance data from my GP records for the purposes of this research study only.	
**7.**	I give permission for my data collected during the study to be looked at by responsible individuals from the **PIMMS-WL** trial office at the University of Birmingham Clinical Trials Unit (BCTU), University of Southampton, from the sponsor, from regulatory authorities or from the NHS Trust, where it is relevant to my taking part in this research. I understand that all information collected will be used for medical research only and that I will not be identified in any way in the analysis and reporting of the results.	
**8.**	I agree to my GP being informed of my participation in the **PIMMS-WL** trial, a copy of my signed consent form to be sent, and my GP to be contacted to obtain any medical history relevant to this study.	
**9.**	I agree to take part in the **PIMMS-WL** trial.	

**OPTIONAL CONSENT:**	**Please initial one box only**	**I agree**	**I do not agree**

**10.**	I am willing for the nurse to audio record the part of my immunisation visits when she discusses the study with me. The recording and transcription will be securely transferred and stored at the University of Birmingham Clinical Trials Unit. The recording will be transcribed by an external company and analysed for the purposes of this research only.	☐	☐

Name of Participant: ……………………………………………………………………………………….

Signature: …………………………………………………………. Date:……………………………………

Name of Person taking consent: …………………………………………………………………………….

Signature: …………………………………………………………. Date:……………………………………

Original to be filed in the Site File; 1 copy for patient; 1 copy to be kept with patient's GP record; 1 copy to be sent to BCTU
Please return a copy of this form to: The PIMMS-WL Trial, Birmingham Clinical Trials Unit (BCTU),
Institute of Applied Health Research, Public Health Building, University of Birmingham, Edgbaston, Birmingham, B15 2TT
