# Supplementary material for: Protocol for the feasibility and acceptability of a brief routine weight management intervention for postnatal women embedded within the national child immunisation programme: randomised controlled cluster feasibility trial with nested qualitative study (PIMMS-WL)
Source: BMJ Open. 2020 Feb 16;10(2):e033027. doi: 10.1136/bmjopen-2019-033027 (PMC7045221; doi:10.1136/bmjopen-2019-033027)
Supplement: Supplementary data [file bmjopen-2019-033027supp004.pdf]

Table 3: STOP-GO traffic light criteria

Green light	• Recruitment rate ≥80% of the target (n=80; i.e. recruit at least 64 women), ≥50% of the intervention group weighs themselves weekly ≥60% of the time and ≥60% of participants have registered with POWeR. If all three criteria are met we will proceed to application for the full trial with the protocol unchanged (unless there is a clear message from the interviews that would improve the protocol).
Amber light	• Recruitment rate of 50-79% of the target (n=80; i.e. recruit between 40 and 63 women), 40-49% of the intervention group weighs themselves weekly 40-59% of the time and 40-59% of the intervention group registered with POWeR. If one or more of our amber light criteria are met, we will plan to adapt the protocol in light of the results of the feedback from the interviews and our experience to improve whichever criteria are not at the “green-light” level before proceeding to a full trial. In discussion with the trial steering committee we will assess whether adaption of the protocol will require further assessment before progressing.
Red light	• Recruitment rate of <50% of the target (n=80; i.e. recruit less than 40 women), <40% of the intervention group weighs themselves weekly 40-59% of the time and <40% of the intervention group have registered with POWeR. If one or more of these criteria are met, we would consider the current protocol not feasible and not progress to a full RCT with the current design. An additional red light criteria would be concerns from the TSC that immunisation rates have been adversely affected.
