# Supplementary material for: Protocol for the feasibility and acceptability of a brief routine weight management intervention for postnatal women embedded within the national child immunisation programme: randomised controlled cluster feasibility trial with nested qualitative study (PIMMS-WL)
Source: BMJ Open. 2020 Feb 16;10(2):e033027. doi: 10.1136/bmjopen-2019-033027 (PMC7045221; doi:10.1136/bmjopen-2019-033027)
Supplement: Supplementary data [file bmjopen-2019-033027supp005.pdf]

Table 4: Qualitative study objectives

1.	To explore whether the child immunisation appointments are an appropriate setting for postnatal mother's weight to be monitored
2.	To capture mother's views on how useful the intervention was at helping them manage their weight
3.	To determine what elements of the intervention facilitated and/or impeded its acceptability
4.	Explore what participants found helpful and unhelpful
5.	To investigate what aspects of the intervention were acceptable and unacceptable to participants and practice nurses, as well as the reasons for these feelings and opinions
6.	To assess what components of the intervention may need to be amended, if any
7.	To assess if the intervention leads to the mothers experiencing any anxiety or psychological harm relating to their weight
8.	To capture nurse's views on the impact of delivering the intervention had on the structure and duration of the child immunisation appointment
