# Supplementary material for: Protocol for the feasibility and acceptability of a brief routine weight management intervention for postnatal women embedded within the national child immunisation programme: randomised controlled cluster feasibility trial with nested qualitative study (PIMMS-WL)
Source: BMJ Open. 2020 Feb 16;10(2):e033027. doi: 10.1136/bmjopen-2019-033027 (PMC7045221; doi:10.1136/bmjopen-2019-033027)
Supplement: Author's manuscript [file bmjopen-2019-033027.draft_revisions.pdf]

BMJ Open is committed to open peer review. As part of this commitment we make the peer review history of every article we publish publicly available.

When an article is published we post the peer reviewers' comments and the authors' responses online. We also post the versions of the paper that were used during peer review. These are the versions that the peer review comments apply to.

The versions of the paper that follow are the versions that were submitted during the peer review process. They are not the versions of record or the final published versions. They should not be cited or distributed as the published version of this manuscript.

BMJ Open is an open access journal and the full, final, typeset and author-corrected version of record of the manuscript is available on our site with no access controls, subscription charges or pay-per-view fees (http://bmjopen.bmj.com).

If you have any questions on BMJ Open's open peer review process please email info.bmjopen@bmj.com

BMJ Open

Protocol for the feasibility and acceptability of a brief routine weight management intervention for postnatal women embedded within the national child immunisation programme: randomised controlled cluster feasibility trial with nested qualitative study (PIMMS-WL)

Journal:	*BMJ Open*
Manuscript ID	bmjopen-2019-033027
Article Type:	Protocol
Date Submitted by the Author:	17-Jul-2019

Complete List of Authors:	Parretti, Helen; University of Birmingham, Insititue of Applied Health Research<br>Ives, Natalie; University of Birmingham, BCTU<br>Tearne, Sarah ; University of Birmingham Clinical Trials Unit<br>Vince, Alexandra ; University of Birmingham Edgbaston Campus, Birmingham Clinical Trials Unit<br>Greenfield, Sheila; University of Birmingham Edgbaston Campus<br>Jolly, Kate; University of Birmingham, Institute of Applied Health Research<br>Jebb, Susan; University of Oxford<br>Frew, Emma; University of Birmingham, Health Economics<br>Yardley, Lucy; University of Southampton, Psychology; University of Bristol, School of Health Sciences<br>Little, Paul; University of Southampton, Primary Care and Population Science;<br>Pritchett, Ruth; University of Birmingham Institute of Applied Health Research<br>Daley, Amanda; Loughborough University, School of Sport, Exercise and Health Sciences

Keywords:	postnatal, weight, QUALITATIVE RESEARCH, randomised cluster trial, Obesity

SCHOLARONE™
Manuscripts

I, the Submitting Author has the right to grant and does grant on behalf of all authors of the Work (as defined in the below author licence), an exclusive licence and/or a non-exclusive licence for contributions from authors who are: i) UK Crown employees; ii) where BMJ has agreed a CC-BY licence shall apply, and/or iii) in accordance with the terms applicable for US Federal Government officers or employees acting as part of their official duties; on a worldwide, perpetual, irrevocable, royalty-free basis to BMJ Publishing Group Ltd ("BMJ") its licensees and where the relevant Journal is co-owned by BMJ to the co-owners of the Journal, to publish the Work in this journal and any other BMJ products and to exploit all rights, as set out in our licence.

The Submitting Author accepts and understands that any supply made under these terms is made by BMJ to the Submitting Author unless you are acting as an employee on behalf of your employer or a postgraduate student of an affiliated institution which is paying any applicable article publishing charge ("APC") for Open Access articles. Where the Submitting Author wishes to make the Work available on an Open Access basis (and intends to pay the relevant APC), the terms of reuse of such Open Access shall be governed by a Creative Commons licence – details of these licences and which Creative Commons licence will apply to this Work are set out in our licence referred to above.

Other than as permitted in any relevant BMJ Author's Self Archiving Policies, I confirm this Work has not been accepted for publication elsewhere, is not being considered for publication elsewhere and does not duplicate material already published. I confirm all authors consent to publication of this Work and authorise the granting of this licence.

For peer review only

Protocol for the feasibility and acceptability of a brief routine weight management intervention for postnatal women embedded within the national child immunisation programme: randomised controlled cluster feasibility trial with nested qualitative study (PIMMS-WL)

Helen M Parretti[1], Natalie Ives[2], Sarah Tearne[2], Alexandra Vince[2], Sheila M Greenfield[1], Kate Jolly[1], Susan A Jebb[3], Emma Frew[1], Lucy Yardley[4,5], Paul Little[6], Ruth V Pritchett[1], Amanda J Daley[7]*

*Corresponding author
Professor Amanda Daley
School of Sport, Exercise and Health Sciences,
Loughborough University,
Loughborough,
LE11 3TU, UK
a.daley@lboro.ac.uk

1 Institute of Applied Health Research, University of Birmingham, Edgbaston, Birmingham, B15 2TT

2 Birmingham Clinical Trials Unit, Public Health Building, University of Birmingham, Edgbaston, Birmingham, B15 2TT

3 Nuffield Department of Primary Care Health Sciences, University of Oxford, Radcliffe Observatory Quarter, Woodstock Road, Oxford, OX2 6GG, UK and Oxford NIHR Biomedical Research Centre.

4 School of Psychological Science, University of Bristol, Priory Road, Clifton BS8 1TU, UK

5 Department of Psychology, Building 44 Highfield Campus, University of Southampton, SO17 1BJ, UK

6 Primary Care and Population Sciences, Aldermoor Health Centre, Aldermoor Close, Southampton, SO16 5ST

7 School of Sport, Exercise and Health Sciences, Loughborough University, Loughborough, LE11 3TU, UK

Main text word count: 4422

EF

For peer review only

ABSTRACT

Introduction

On average women retain 5-9kg of weight 1 year after giving birth which can significantly increase the risk of later obesity and chronic diseases. Previous trials in this population have been effective in reducing weight, but they are too intensive and costly to deliver at scale in routine care. There is a need for low-cost interventions to facilitate weight loss in this population of women.

Methods and analysis

The primary aim of this study is to assess the feasibility of delivering a weight management intervention for overweight/obese postnatal women within child immunisation appointments. We will conduct a randomised controlled cluster feasibility trial with a nested qualitative study to assess study recruitment and acceptability of the intervention. GP practice (cluster) will be the unit of randomisation, with practices randomised to offer usual care plus the intervention or usual care only. 80 women will be recruited.

The intervention group will be offered brief support that encourages self-management of weight when they attend child immunisation appointments. Practice nurse will encourage women to weigh themselves weekly and record this, and to make healthy lifestyle choices through use of an online weight management programme. Women will be advised to aim for 0.5-1kg weight loss per week. At each child immunisation appointment a nurse will assess progress against this goal by weighing women. The comparator group will receive a healthy lifestyle leaflet.

Data on weight, body fat, depression, anxiety, body-image, eating behaviours and physical activity will be collected at baseline and three months. Women and nurses will be interviewed to ascertain their views about the intervention. The decision to proceed to the phase III trial will be based on pre-specified stop-go criteria.

Ethics and Dissemination

Results will inform the design of phase III randomised controlled trial. The National Research Ethics Committee approved the study protocol.

Trial Registration number: ISRCTN12209332

For peer review only

EF

Strengths and limitations of this study:

- This is the first study to assess the feasibility of embedding a weight management intervention for postnatal women within usual care contacts in primary care.
- This study uses mixed methods and will draw on both quantitative and qualitative data to assess the feasibility and acceptability of the intervention.
- The intervention is based on self-regulation theory with external accountability.
- Rather than rely on self-reports, the study uses objective recordings from real time weighing scales to assess the frequency of self-weighing.

For peer review only

Key Words: postnatal, randomised cluster feasibility trial, obesity, weight, qualitative

For peer review only - http://bmjopen.bmj.com/site/about/guidelines.xhtml

INTRODUCTION

Pregnancy is associated with significant weight gain and many women report pregnancy as the critical point for the onset of excess weight. (1-2) The postnatal period is characterised not only by weight retention, but also susceptibility to further weight gain, which can significantly increase the risk of later obesity and serious chronic diseases.(3-5) There is also an association between postnatal weight retention and poor mental health.(6-8) It is estimated that on average women gain about 14-15kg during pregnancy and at 1 year after birth 5-9kg is retained.(2-3, 9-10) Of women who are overweight prior to conception, 44% are living with obesity by 1 year after giving birth, while 97% of women who are living with obesity prior to pregnancy remain so at 1 year postnatally.(5) Therefore, there is a need to intervene routinely in the postnatal period, to help women manage their weight after having a baby and minimise long-term health risks.

Several systematic reviews have identified the need for high quality trials on how to best help women to lose weight after having a baby.(11-16) Within these reviews, the vast majority of studies have recruited small sample sizes and evaluated intensive physical activity and/or dietary interventions delivered by specialists; while these types of interventions can be effective, they cannot be delivered to all the women who give birth each year in the UK, of whom nearly two-thirds will be living with overweight.(17) A recent systematic review of reviews of lifestyle interventions for postnatal weight management highlighted the lack of studies that have tested an intervention embedded within routine health care.(16)

Self-management strategies and weight loss

. A systematic review of randomised controlled trials (RCTs) to determine the effectiveness of self-help interventions for weight loss (18), has shown that self-help interventions can lead to small, but significantly greater weight loss than unsupported attempts to lose weight at six months compared with minimal interventions. In the small group of studies providing data at 12 months, weight loss was no longer significant, suggesting that self-help interventions on their own may not be useful for sustaining long term weight loss, and that additional components, within weight loss interventions are required. Nonetheless, given the potential scalability and relatively low cost of this type of intervention, self-help programmes may be a useful component within a broader intervention to treat those who are overweight.

Self-monitoring of weight by regular weighing is common to many self-management interventions. The potential efficacy of regular weighing is based on the principles of self-regulation theory.(19-20) Self-regulation theory describes three distinct stages; self-monitoring; self-evaluation and self-reinforcement. Self-monitoring is a method of systematic self-observation, periodic measurement

EF

For peer review only - http://bmjopen.bmj.com/site/about/guidelines.xhtml

and recording of target behaviours with the goal of increasing self-awareness. The awareness fostered during self-monitoring is an essential initial step in promoting and sustaining behaviour change. Strong evidence supports the role of self-monitoring as an effective strategy in the health behaviour change process. Reviews by Michie *et al.* of effective behavioural techniques for healthy eating, physical activity and reduction of alcohol consumption concluded that self-monitoring was effective alone, but when combined with other techniques the effect size nearly doubled.(21-22) In a systematic review of RCTs to examine the effectiveness of self-weighing as a strategy for weight loss, multi-component interventions including self-weighing compared to no/minimal control also resulted in mean differences of -3.7 kg (95% CI -4.6 to -2.9).(23)

Accountability/audit and feedback

There has been some evidence to suggest that adding accountability to a self-weighing programme increases weight loss.(23) In a review by Madigan *et al.*, in 13 of the included trials, the intervention group asked to weigh themselves knew that they were accountable to a therapist/researcher, while this was not the case in two trials. There was a significant difference in mean weight loss between intervention and control for those trials with accountability compared with those without (-4.0kg, 95% CI -5.0 to -3.0kg versus -2.3kg, 95% CI -3.1 to -1.5kg; p=0.007).(23) Participants in group weight loss programmes often report that it is the weekly weigh-in that is the most salient component of the programme, providing external accountability and keeping them committed to their diet and physical activity plan.(24) Based on this evidence, it can be hypothesised that including accountability/audit to self-help and self-monitoring interventions could further facilitate weight loss.

Internet-based weight management interventions

The widespread use of technology has led to an interest in digital health interventions, including for weight management. Systematic reviews of eHealth or web-based interventions for weight loss and/or maintenance in adults have found that these interventions can result in modest weight loss in individuals with overweight or obesity.(25-26) One such digital interventions is the POWeR online weight management programme.(27) The POWeR programme was designed for use with minimal additional support and was developed with extensive user feedback; the development process and content of the POWeR intervention is described elsewhere. (28-29) The POWeR programme has been shown to result in significant weight loss at 12 months in primary care patients with overweight when combined with brief nurse support.(27) It focuses principally on fostering users' self-regulation skills for autonomously self-managing their weight, rather than providing detailed dietetic advice. A recent systematic review concluded that internet-based weight management interventions for postnatal mothers appear to be beneficial, and survey study results have reported

For peer review only - http://bmjopen.bmj.com/site/about/guidelines.xhtml

that irrespective of age or socio-economic status, 90.5% of new mothers use the internet as a source of information with weight loss as the fourth most searched topic (20-31). Therefore, based on this evidence it may be hypothesised that including an online intervention may be beneficial for postnatal weight loss.

Study rationale

There is a need to support women to lose excess weight in the postnatal period. Given the large numbers of women who give birth each year either with pre-existing overweight or obesity, or who gain excess weight during this period, an intervention is needed that can be delivered at scale without significant implications for a healthcare workforce. In addition, the National Institute for Health and Care Excellence (NICE) has highlighted the low quality of previous research as a limitation to developing clinical guidance in this area.(32) Here we investigate the feasibility of a postnatal weight management intervention embedded within pre-existing healthcare contacts. This pragmatic intervention is based on existing evidence for the effectiveness of both self-monitoring and an online programme for weight management (POWeR).(19, 27)

Aims and objectives

The primary objective is to produce evidence that a large-scale phase III cluster RCT of a weight management intervention embedded in the national child immunisation programme (33) would be acceptable to women and feasible to conduct. Detailed objectives are listed in Supplementary Table 1. This protocol is reported according to the Standard Protocol Items Recommendations for Interventional Trials (SPIRIT) guidelines.(34)

METHODS AND ANALYSIS

Trial Design

PIMMS-WL is a feasibility cluster RCT to assess the feasibility and acceptability of a weight management intervention, based on self-regulation theory, in women who have recently given birth.(20) This will be assessed through a composite of quantitative and qualitative data. Key stages of the study are shown in Figure 1.

Trial Setting

This study will take place in Birmingham, UK between 01/09/2017 and 31/10/2019. The initial identification of women will take place within Birmingham Women and Children's NHS Foundation Trust and within participating GP practices, and the trial will take place within GP practices around the West Midlands. The baseline and follow-up assessments will be completed in participants' homes.

For peer review only - http://bmjopen.bmj.com/site/about/guidelines.xhtml

Study participants

Women will be eligible if they are aged ≥18 years; are at least four weeks postnatal; have a BMI ≥25kg/m^2 at the time of recruitment; are able to provide written informed consent and do not meet any of the exclusion criteria (Supplementary Table 2). G that the intervention is embedded in immunisation appointments to be eligible women need to have not yet attended their child's first immunisation appointment and to be planning to have their child immunised.

Consent

Written informed consent into the PIMMS-WL trial will be a two stage process conducted by a researcher at the baseline home visit (see Appendix 1 for Informed Consent Forms). First, written informed consent will be sought for screening to confirm all eligibility criteria are met. Once all eligibility criteria have been confirmed, participants will be invited to give their written informed consent to be enrolled into the RCT.

Sample Size and Recruitment

This is a feasibility trial therefore a formal sample size calculation has not been conducted. The trial is not designed or powered to detect a statistically significant difference in effectiveness between the two treatment groups.

We will recruit 10-12 GP practices (clusters) across Birmingham, UK and the list of participating practices will be held by Birmingham Clinical Trials Unit (BCTU). We will then aim to recruit 80 participants from these GP practices over eight months. Potentially eligible participants will be identified initially from medical records at Birmingham Women's and Children's NHS Foundation Trust (BWCH). Initial screening of medical records by BWCH will confirm the women is aged ≥18 years, has given birth at least four weeks previously, and is registered at a participating GP practice. We will exclude mothers whose babies have died or have been removed from their care.

A trial invitation letter and participant information sheet (PIS) will be mailed to potentially eligible women by BWCH, which includes a telephone number to call or a reply slip to return if they are interested in participating. Interested women will undergo telephone screening for eligibility. For women who fulfil the initial screening criteria and are still interested in taking part in the trial, a baseline home visit with a researcher will be organised.

Some additional recruitment strategies may also be used if required:

- posters and flyers advertising the study will be displayed in waiting rooms at participating GP practices.

For peer review only - http://bmjopen.bmj.com/site/about/guidelines.xhtml

- participants may be informed about the trial directly at appointments with the GP or other health care professional post-delivery and prior to the 2-month immunisation appointment in participating GP practices
- a researcher may be available at the GP practice on specific clinic dates. The PIMMS-WL researcher will provide potential participants with the letter of invitation and PIS directly either to read at the GP practice or for the participant to take home.

If, after having read the letter of invitation and PIS, a woman is interested in participating, they will contact the PIMMS-WL research team for telephone screening (or be screened by the PIMMS-WL researcher at the GP practice) and a baseline home visit will be arranged as described above.

For peer review only

Randomisation

The unit of randomisation is the GP practice (cluster). GP practices will be randomised in a 1:1 ratio to provide the usual care plus a weight management intervention or usual care only (comparator group) using minimisation for GP practice list size (large: 6000 or more; small: under 6000 patients) and Index of Multiple Deprivation (IMD) rank score.(35) The IMD will be based on the postcode of the GP practice; the IMD rank score ranges from 1 to 32,844 and will be divided into tertiles of high, medium and low levels of deprivation. BCTU will create the randomisation list to allocate GP practices to the two trial groups.

Trial intervention (usual care plus weight management group)

The intervention will be delivered within the national child immunisation programme to minimise costs and additional burden on the NHS workforce and avoid the need for additional contacts with health professionals at this busy time in women's lives. In the first year, babies are routinely immunised at two, three, four and twelve months of age; we plan to embed the intervention within these pre-existing immunisation contacts. The intervention group will be offered brief support that encourages active self-management of weight in the postnatal period when they attend their practice to have their child immunised during the first year of life. The intervention involves motivation and support by nurses to encourage women in the postnatal period to make healthier lifestyle choices through self-monitoring of weight and signposting to an online weight management programme (POWeR).(27)

Women will be provided with weighing scales and asked to weigh themselves weekly. The intervention will be delivered until the third immunisation appointment. Women will be advised and encouraged to record their weight on a weight record card that will be attached to the child health record (the 'red book') and also within the POWeR online programme.(27). The 'red book' includes a record of infant examinations and investigations, growth and immunisation history.

For peer review only - http://bmjopen.bmj.com/site/about/guidelines.xhtml

Weight loss goals

No clinical guidelines exist for rates of healthy weight loss for postnatal women, but for the general population NICE recommend 0.5-1kg per week.(36) Women will be advised to aim for 0.5-1kg per week weight loss until they have achieved a BMI less than 25 kg/m^2 and are no heavier than their self-reported pre-pregnant weight.

Accountability

Practice nurses will weigh the women at each child immunisation visit and record this weight as a source of regular external accountability. They will not provide any weight management advice.

Online weight loss programme (POWeR: Positive Online Weight Reduction)

Nurses will signpost women to the POWeR online weight loss programme for support and assistance with goal setting, action planning and implementation of changes to their lifestyle. Information about breast feeding and weight loss (such as diet, physical activity and rate of recommended weight loss) will be added to the programme for the purpose of this trial. Women will be encouraged to use the website weekly to track their weight, set and review eating and physical activity goals, and receive personalised advice.

Training of practice nurses

All nurses who deliver child immunisations at intervention practices will be trained to deliver the intervention following a standard protocol. A training manual will be provided, with instructions for the tasks to be undertaken by nurses during immunisation appointments.

Intervention fidelity

Two immunisation/intervention consultations per nurse will be audio recorded so that intervention fidelity can be assessed against an intervention checklist criteria, and the length of time taken to deliver the intervention monitored.

Usual care group

Women enrolled into the trial and registered at GP practices in the usual care comparator group will receive brief written information about following a healthy lifestyle using the NHS EatWell guide leaflet.(37)

Masking

It is not possible to mask participants or those providing the intervention to group allocation. In this feasibility study, it is also not possible to mask the outcome assessor, as the same member of the PIMMS-WL research team will need to undertake both the baseline and follow-up home visit to collect data. We do not believe this will introduce bias, as the aim of this study is to assess the

For peer review only - http://bmjopen.bmj.com/site/about/guidelines.xhtml
For peer review only

feasibility of undertaking a large phase III cluster RCT, and these outcomes are not affected by knowledge of group allocation and the data relating to the feasibility outcomes are not collected during the home visits. The proposed primary outcome for the phase III trial is weight which is objectively measured using scales.

Baseline assessment and follow-up

Baseline visit

The baseline visit will occur between 6-7 weeks postnatally and before the first immunisation visit.

Follow-up visit

A follow-up visit will take place three months post baseline. Women in the intervention group will be invited to participate in the qualitative study interview (see below). A £20 gift voucher will be given to participants as reimbursement for any inconvenience and expenses incurred.

Patient and public Involvement

Members of the CLAHRC-WM maternity patient and public involvement (PPI) group (PRIME) provided input into the development of this study. They gave advice on the relevance of the study question based on their experiences and their assessment of the burden of the intervention on potential participants. Two members of the public will be invited to join the Trial Steering Committee (TSC). The PPI group will meet three times a year and will provide input into the topic guide for the qualitative interviews and have input into and review all patient facing documents. They will review all dissemination materials and be involved in the dissemination of the trial results.

Primary outcome

The primary outcome is the feasibility of undertaking a full-scale phase III cluster RCT, and will include assessment of the following:

- whether the trial is appealing to women (via assessment of the recruitment rate to ensure a full phase III trial is feasible);
- whether the intervention is acceptable;
- whether the intervention has any adverse impact on infant immunisation rates;
- the number of women who complete the trial and complete the trial questionnaires.

The decision to proceed will be based on pre-specified stop-go criteria (see Supplementary Table 3).

Outcome data

Outcome data will be collected at baseline and three months follow-up (except height (baseline only)). While this feasibility trial will not be powered to detect meaningful differences in outcome

For peer review only - http://bmjopen.bmj.com/site/about/guidelines.xhtml

measures, it will give us the opportunity to ensure that there are no issues with the completion of these measures in a main trial. Height and weight will be measured at baseline to calculate BMI. Weight and percentage body fat will be assessed using a Tanita SC-240MA analyser using a standardised procedure (e.g. light clothing). Demographic (e.g. age, ethnicity, socioeconomic status), lifestyle and pregnancy related information (e.g. history of pregnancy related problems, mode of delivery), infant feeding practices, and maternal sleeping patterns will be collected. Anxiety and depression will be assessed using the Hospital Anxiety and Depression Scale, body image using the Body Image State Scale, eating habits using the three-factor eating questionnaire and self-reported physical activity will be measured using the Pregnancy Physical Activity Questionnaire.(38-41)

At follow-up, self-weighing will be assessed using the Daily Self-Weighing Perceptions Questionnaire and weight control strategies using the Weight Control Strategies Scale.(42) We will also record whether the usual care group have accessed the POWeR website to assess any intervention contamination. To inform the design of the economic evaluation in the phase III trial, we will explore the acceptability of the ICECAP instrument (43), a broader measure of health than the EQ-5D (44) that focuses on general wellbeing. This will be done by assessing rates of completion of the ICECAP, and incorporating questions about the acceptability of the ICECAP into the qualitative interviews.

Eating behaviours (both groups)

Our hypothesis is that regular self-weighing and monitoring of weight leads to the development of self-regulation and conscious cognitive energy restraint.(20,45) Using the revised three-factor eating questionnaire (41), we will examine if feedback from self-weighing has led to development of conscious cognitive energy restraint of eating. We will also measure uncontrolled eating and emotional eating.

Weight control strategies (both groups)

Weight control strategies will be assessed at follow-up in both groups using the Weight Control Strategies Scale. As well as a total score, the domains of dietary choices, self-monitoring strategies, physical activity and psychological coping are measured.(42)

Objective assessment of self-weighing/adherence (intervention group)

As an objective measure of compliance, the intervention group will be given weighing scales (BodyTrace, USA) that objectively record weight every time participants weigh themselves and then this information is remotely transmitted back to the research team at the University of Birmingham. These scales are only included as an objective process measure of adherence to self-weighing and

For peer review only - http://bmjopen.bmj.com/site/about/guidelines.xhtml
For peer review only

we will not provide any feedback to participants, nor will we monitor changes in weight during the trial follow-up period.

Weight record cards (intervention group)

The intervention group will complete weight record cards and we will collect these from participants as a measure of intervention implementation at the follow-up visit. The record cards will also tell us how much of the intervention was delivered by practice nurses per protocol.

Perceptions of regular self-weighing (intervention group)

In the intervention group, we will use five items from Steinberg's Daily Self-Weighing Perceptions Questionnaire (46) to measure perceptions of regular monitoring of weight at follow-up.

Use of the POWeR online programme (intervention group)

Using participants' unique usernames POWeR automatically records participants' usage of the website (e.g. pages visited, when/for how long, goals and weights entered and dates).

Attendance at immunisation appointments (both groups)

To ensure the intervention does not have an adverse impact on women attending the immunisation appointments all practices will be asked to provide data on all immunisation appointments attended by both groups. Any missed appointments will be investigated and a reason allocated by the GP surgery. We will also collect patient reported attendance at the immunisation appointments at the follow-up visit.

Assessment of harms

We will not be collecting adverse events for this trial. Serious Adverse Events (SAEs) which are attributable to the trial intervention will be documented and reported from the date of consent into the PIMMS-WL study until 30 days after the three month follow-up visit. SAEs or deaths related to pre-existing medical conditions are not considered related to the trial intervention and are therefore excluded from notification as SAEs.

Withdrawal

Participants will be aware that they can freely withdraw from the trial (or part of) at any time. Details of withdrawal will be clearly documented.

If a recruited GP practice withdraws from participation in the trial prior to randomisation, they may be replaced by another practice. If a practice withdraws after randomisation, consideration will be given to replacing with another practice.

Statistical analysis

For peer review only - http://bmjopen.bmj.com/site/about/guidelines.xhtml

A separate statistical analysis plan will provide a more comprehensive description of the planned analyses. A brief outline of the planned analyses in relation to the STOP-GO criteria is provided below. The data analysis for this feasibility trial will be descriptive and mainly focus on confidence interval estimation, with no hypothesis testing performed.

The recruitment rate will be presented as a percentage based on the number of participants who took part in the trial divided by the target recruitment (n=80). BWCH will also provide data on the number of invitation letters sent, along with data on the age and ethnicity (in summary format) of the women who were sent an invitation letter.

The quantitative assessment of whether the intervention is acceptable will be based on the adherence of women to weekly self-weighing and use of the online POWeR programme. In the first instance, the objective recording of weight on the scales will be used to assess the frequency of self-weighing/adherence.

In addition, to check that the intervention has no adverse impact on infant immunisation rates, the proportion of babies who attended all three immunisation appointments will be reported for each practice (both groups) and the rate compared with the normal immunisation rate for the practice. Reasons for missed appointments will be summarised descriptively. Recruitment, adherence and immunisation rates will be summarised as proportions with 95% confidence intervals.

Qualitative study

We will use semi-structured interviews after trial follow-up to explore the views of women about the intervention and experiences of the practice nurses delivering the intervention.(47) Topic guides will be informed by existing literature.

We will purposively sample up to 15 women (*e.g.* age, ethnicity, socioeconomic status and BMI category at enrolment) and 8-10 practice nurses and ask them about their experiences of the trial, to allow for theme saturation to be reached. (48-49) Interviews will be conducted using a topic guide which will explore the women's experiences of and attitudes to trial participation, their understanding and rating of the importance of postnatal weight management, strategies used for weight management and their use of the online weight programme. For practice nurses, we will explore their experiences of delivering the intervention, barriers and facilitators, the content and phrasing of discussions with women about weight and views about the approaches that work best and least well. The objectives of the qualitative study are summarised in Supplementary Table 4.

It is anticipated that interviews will take place face-to-face, but it is possible for logistical reasons that some will need to take place by telephone. Interviews will be recorded and transcribed with the

For peer review only - http://bmjopen.bmj.com/site/about/guidelines.xhtml
For peer review only

permission of participants and thematically analysed using the Framework Method.(50) Data management will be facilitated by the use of QSR NVivo 11.(51) A list of overall and individual themes for women and practice nurses will be compiled to allow for cross group/individual comparison. Data collection and analysis will be iterative. All digitally recorded data that is collected during interviews will be recorded on an encrypted audio recording device. The transcriptions will be anonymised.

Decision to progress to the phase III trial

For the phase III trial to take place there needs to be evidence from this feasibility trial of meeting pre-specified STOP-GO rules. The trial is too small to include meaningful and sensitive STOP-GO criteria regarding the impact of the intervention on immunisation rates. However, we will check that the intervention has not adversely affected usual immunisation rates at each GP practice (as described above). The criteria to proceed to the phase III trial will be based on three criteria: recruitment rate; adherence to weekly self-weighing and registration with the online weight loss programme (POWeR) using the traffic light system shown in Supplementary Table 3.

Data collection and management

All data will be entered onto the PIMMS-WL trial database. All trial paperwork will be stored in lockable filing cabinets in a secure, swipe access part of the University of Birmingham. Password protected electronic databases, on secure University of Birmingham servers for trial data will have access limited to BCTU members of staff working on the trial.

ETHICS AND DISSEMINATION

Ethics

The study will be conducted according to the principles of the Declaration of Helsinki and in accordance with other relevant national guidelines, regulations, and acts. The trial has been granted research ethics committee approval by the West Midlands and Black Country National Research Ethics Committee (17/WM/0399, Protocol V9.0, 30/6/19).

Monitoring and oversight

The trial will form part of a portfolio of studies hosted and managed by BCTU. The University of Birmingham holds the relevant insurance for this study and is the nominated sponsor for this study.

As this is a feasibility trial, we do not propose to hold a Data Monitoring and Ethics Committee. A TSC will provide overall supervision and oversight of the trial and ensure it is conducted in accordance with the principles of good clinical practice and relevant regulations. The TSC agreed the trial protocol and will agree to any protocol amendments. The TSC will include members who are

For peer review only - http://bmjopen.bmj.com/site/about/guidelines.xhtml

independent of the investigators, their employing organisations, funders and sponsors. The TSC will monitor trial progress and conduct and advise on scientific credibility. An explicit purpose of the TSC is to monitor the immunisation uptake rates at the GP practices.

Dissemination

A lay summary of the study is available on the National Institute for Health Research website (https://bepartofresearch.nihr.ac.uk/trial-details/trial-detail?trialId=17314&location=&distance=). Final results of this feasibility study will be made publicly available through publication in peer-reviewed journals and presented at relevant conferences.

DISCUSSION

The postnatal period is characterised not only by weight retention, but also by susceptibility to further weight gain. Most trials in this population include small sample sizes and/or evaluated intensive weight loss interventions. These types of interventions cannot be offered to all women who give birth in the UK every year. This study will generate evidence as to whether a full phase III trial to test the effectiveness of this novel postnatal weight management intervention embedded within existing primary care contacts is feasible. Currently there is no national guidance for health professionals regarding postnatal weight management and the full trial testing the effectiveness of this intervention would have the potential to influence policy and future national guidelines.

DECLARATIONS

Competing interests

The authors declare that they have no competing interests.

Funding

This work is supported by the National Institute of Health Research (NIHR) Health Technology Assessment programme grant number 15/184/14. HMP is funded by a National Institute for Health Research Academic Clinical Lectureship.

Authors' contributions

AJD developed the original idea for the study along with HMP, KJ, SJ and RP. LY and PL contributed to the protocol in relation to digital technology. EF was responsible for writing the health economics aspects of the protocol. SG was involved in developing the qualitative component of the study protocol. NR drafted the paper with input from all authors. ST and AV further contributed to the

EF

For peer review only - http://bmjopen.bmj.com/site/about/guidelines.xhtml

development of the protocol after funding was awarded. All authors read, commented and approved the final manuscript.

Acknowledgements

The authors would like to thank the lay representatives who contributed to the trial steering committee and the lay representatives who contributed to the study design.

Disclaimer

This report is independent research commissioned by the National Institute for Health Research. The views expressed in this publication are those of the authors and not necessarily those of the NHS, the National Institute for Health Research or the Department of Health.

Ethics approval

This manuscript is based on Protocol V9.0 dated 30/6/19–submitted and approved by the West Midlands and Black Country NHS Research Ethics Committee (17/WM/0399, approval date xxxx).

For peer review only

For peer review only - http://bmjopen.bmj.com/site/about/guidelines.xhtml

REFERENCES

1. Gore SA et al. The role of postpartum weight retention in obesity among women: a review of the evidence. Ann Behav Med 2003;26:149.
2. Walker LO. Managing excessive weight gain during pregnancy and the postpartum period. J Obstet Gynecol Neonatal Nurs 2007;36:490.
3. Walker LO et al. Do low income women attain their pre-pregnant weight by the 6th week of postpartum? Ethn Dis 2004;14:119.
4. Schmitt NM et al. The association of pregnancy and the development of obesity - results of a systematic review and meta-analysis on the natural history of postpartum weight retention. Int J Obes 2007;31:1642.
5. Prospective Studies Collaboration et al. Body-mass index and cause-specific mortality in 900 000 adults: collaborative analyses of 57 prospective studies. Lancet 2009;373:1083.
6. Beck C. The effects of postpartum depression on maternal-infant interactions: A meta analysis. Nurs Res 1995;44:298.
7. Cooper PJ, et al. Course and recurrence of postnatal depression. Br J Psychiatry 1995;166: 191-5.
8. Murray L. The impact of postnatal depression on infant development. J Child Psychol Psychiatry 1992;33:543-561.
9. NHS Information Centre for Health and Social Care. Statistics on obesity, physical activity and diet: England, 2011. Available at: http://data.gov.uk/dataset/statistics-on-obesity-physical-activity-and-diet-england-2011.
10. Althuizen E, et al. Postpartum behaviour as predictor of weight change from before pregnancy to one year postpartum. BMC Public Health 2011; 11:165.
11. Hoedjes M et al. Effect of postpartum lifestyle interventions on weight loss, smoking cessation and prevention of smoking relapse: a systematic review. Obstet Gynecol Surv 2010;65:10.
12. Fowles ER et al. Postpartum health promotion interventions: a systematic review. Nurs Res 2012;61:296.
13. van der Pligt P et al. Systematic review of lifestyle interventions to limit postpartum weight retention: implications for future opportunities to prevent maternal overweight and obesity following childbirth. Obes Rev. 2013;14:792.
14. Nascimento SL et al. The effect of physical exercise strategies on weight loss in postpartum women: a systematic review and meta-analysis. Int J Obes 2014;38:626.
15. Amorim Adegboye AR et al. Diet or exercise, or both, for weight reduction in women after childbirth. Cochrane Database of Syst Rev 2013.

EF

For peer review only

16. Ferguson JA et al. Behavioural weight management interventions for postnatal women: A systematic review of systematic reviews of randomized controlled trials. Obes Rev 2019: doi.org/10.1111/obr.12834.
17. Office for National Statistics. Annual Mid-year Population Estimates, 2011 and 2012. Available at: http://www.ons.gov.uk/ons/rel/pop-estimate/population-estimates-for-uk--england-and-wales--scotland-and-northern-ireland/mid-2011-and-mid-2012/stb---mid-2011---mid-2012-uk-population-estimates.html#tab-Key-Points.
18. Hartmann-Boyce J et al. Self-help interventions for weight loss in overweight and obese adults: systematic review and meta-analysis. Am J Public Health 2015;105:3.
19. Kanfer FH. Self-monitoring: methodological limitations and clinical applications. J Consult Clin Psychol 1970;35:148.
20. Cameron LD et al. The self-regulation of health and illness behavior. London: Routledge; 2003.
21. Michie S et al. Effective techniques in healthy eating and physical activity interventions: a meta-regression. Health Psychol 2009;28:690.
22. Michie S et al. Identification of behaviour change techniques to reduce excessive alcohol consumption. Addiction. 2012;107:1431.
23. Madigan C et al. Is self weighing an effective weight management tool: systematic review and meta analysis. I J Beh Nut Phys Act 2015 21;12:104.
24. Ahern AL et al. Participants' explanatory model of being overweight and their experiences of 2 weight loss interventions. Ann Fam Med 2013;11:251.
25. Hutchesson MJ et al. eHealth interventions for the prevention and treatment of overweight and obesity in adults: a systematic review with meta-analysis. Obes Rev. 2015;16(5):376.
26. Kozak AT et al. Technology-based interventions for weight management: current randomized controlled trial evidence and future directions. J Behav Med. 2017;40(1):99.
27. Little P et al. An internet-based intervention with brief nurse support to manage obesity in primary care (POWeR+): a pragmatic, parallel-group, randomised controlled trial. Lancet Diabetes Endocrinol 2016; 4(10): 821.
28. Bradbury K et al. Using mixed methods to develop and evaluate an online weight management intervention. Br J Health Psychol. 2015; 20(1):45.
29. Yardley L et al. Integrating user perspectives into the development of a web-based weight management intervention. Clin Obes. 2012; 2(5-6):132.
30. Slomian J et al. The internet as a source of information used by women after childbirth to meet their need for information: A web-based survey. Midwifery. 2017;48:46.

For peer review only

For peer review only - http://bmjopen.bmj.com/site/about/guidelines.xhtml

31. Sherifali D et al. The Effectiveness of eHealth Technologies on Weight Management in Pregnant and Postpartum Women: Systematic Review and Meta-Analysis. J Med Internet Res. 2017;19(10):e337
32. National Institute for Health and Care Excellence. Public Health Guidance 27: Weight management before, during and after pregnancy: costings report. Available at: http://www.nice.org.uk/guidance/ph27/resources/ph27-weight-management-before-during-and-after-pregnancy-costing-report2.
33. Public Health England. Vaccine coverage and cover. Available at: https://www.gov.uk/government/collections/vaccine-uptake.
34. Chan A-W et al. SPIRIT 2013 Statement: Defining standard protocol items for clinical trials. Ann Intern Med 2013;158:200-207.
35. McLennan D et al. The English Indices of Deprivation 2010. Department for Communitites and Local Government. London. Available at: https://www.gov.uk/government/uploads/system/uploads/attachment_data/file/6320/1870718.pdf.
36. National Institute for Health and Care Excellence. Clinical Guidance189: Obesity: identification, assessment and management. Available at: https://www.nice.org.uk/guidance/cg189.
37. Public Health England. Eatwell Guide. Available at: https://www.gov.uk/government/publications/the-eatwell-guide
38. Zigmond AS, Snaith RP. The Hospital Anxiety and Depression Scale. *Acta Psychiatr Scand.* 1983, 67(6):361-70.
39. Chasan-Taber L et al. Development and validation of a pregnancy physical activity questionnaire. Med Sci Sports Exerc 2004;36:1750.
40. Cash TF et al. Beyond body image as a trait: the development and validation of the body image states scale. Eat Disord 2002;10:103.
41. de Lauzon B et al. The Three Factor Eating Questionnaire-R18 is able to distinguish among different eating patterns in a general population. J Nutr 2004;134:2372.
42. Pinto AM et al. Development and validation of the weight control strategies scale. Obesity 2013;21:2429.
43. Al-Janabi H et al. Development of a self-report measure of capability wellbeing for adults: the ICECAP-A. Qual Life Res 2012;21:167.
44. EuroQol Group. EuroQol--a new facility for the measurement of health-related quality of life. Health Policy 1990;16:199.
45. Butryn ML et al. Consistent self monitoring of weight: a key component of successful weight loss maintenance. Obesity 2007:15;3091.

For peer review only - http://bmjopen.bmj.com/site/about/guidelines.xhtml

46. Steinberg DM et al. The efficacy of a daily self-weighing weight loss intervention using smart scales and email. Obesity 2013;21:1789.

47. Jamshed S Qualitative research method-interviewing and observation J Basic Clin Pharm 2014; v.5(4).

48. Lawrence A et al. Purposeful sampling for qualitative data collection and analysis in mixed method implementation research Adm Policy Ment Health. 2015; 42(5): 533.

49. Morse JM. Determining sample size. *Qual Health Res* 2000;10;1:3.

50. Gale NK et al. Using the framework method for the analysis of qualitative data in multi-disciplinary health research *BMC Med Res Methodol* 2013**13**:117

51. QSR International Pty Ltd. NVIVO Qualitative data analysis software V.11. 2012.

For peer review only

EF

Figure and tables

Figure 1: Study flow diagram

Supplementary Table 1: Detailed study objectives

Supplementary Table 2: Inclusion and exclusion criteria

Supplementary Table 3: STOP-GO traffic light criteria

Supplementary Table 4: Qualitative study objectives

For peer review only

For peer review only - http://bmjopen.bmj.com/site/about/guidelines.xhtml

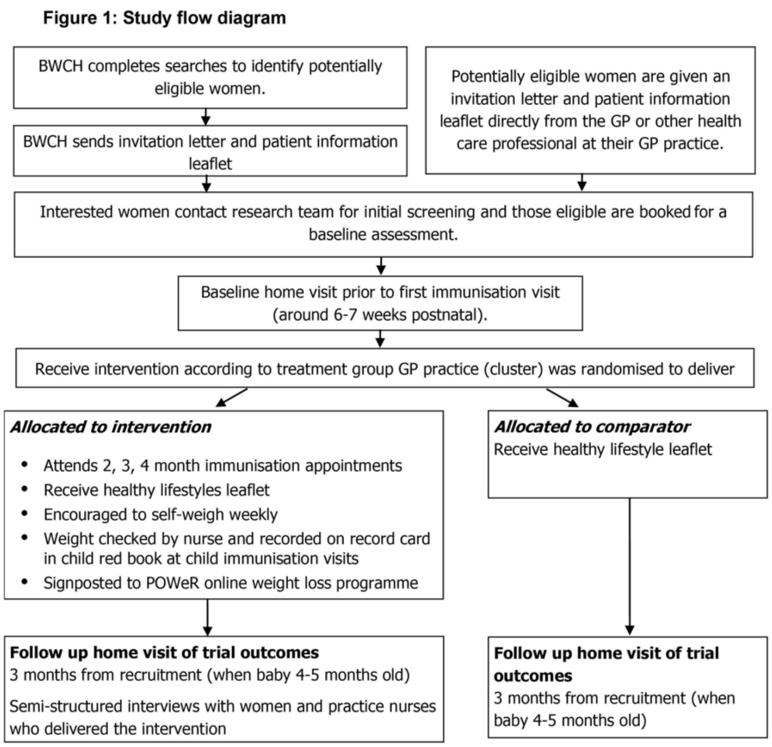

90x90mm (600 x 600 DPI)

Appendix: Informed Consent Forms

PIMMS-WL

UNIVERSITY OF BIRMINGHAM

The PIMMS-WL Trial

Feasibility and acceptability of a brief routine weight management intervention for postnatal women embedded within the national child immunisation programme in primary care: randomised controlled cluster feasibility trial with nested qualitative study.

Patient Screening Consent Form
Version 4.0 1st February 2018

**PIMMS-WL** Trial No:			-					Patient Initials:		

Please initial each box to indicate your consent

**1.**	I confirm that I have read and understood the information sheet for the **PIMMS-WL** trial (Version __, dated DD/MM/YYYY). I have had the opportunity to consider the information, ask questions, and have had these answered satisfactorily.	
**2.**	I understand that my participation is voluntary, that I am not obliged to take part in the full **PIMMS-WL** trial, and the standard of my medical care or legal rights will not be affected.	
**3.**	I understand that data collected that identifies me by name, including my contact details and my consent form, will be transferred from where it is collected and stored at the University of Birmingham Clinical Trials Unit (BCTU). I agree to the transfer and storage of this data for use in the **PIMMS-WL** trial.	
**4.**	I give permission for my data collected during the study to be looked at by responsible individuals from the **PIMMS-WL** trial office at the University of Birmingham Clinical Trials Unit (BCTU), from the sponsor, from regulatory authorities or from the NHS Trust, where it is relevant to my taking part in this research. I understand that all information collected will be used for this research only and that I will not be identified in any way in the analysis and reporting of the results.	
**5.**	I agree to take part in screening in order to determine my eligibility for participation for the **PIMMS-WL** study and I understand that I may not be suitable to take part as detailed in the information sheet.	

Name of Participant: ……………………………………………………………………………………….

For peer review only

Signature: ………………………………………………………. Date:…………………………………

Name of Person taking consent: ……………………………………………………………………….

Signature: ………………………………………………………. Date:…………………………………

Original to be filed in the Investigator's Site File; 1 copy for patient;
1 copy to be kept with patient's GP record; 1 copy to be sent to BCTU
Please return a copy of this form to: The PIMMS-WL Trial, Birmingham Clinical Trials Unit (BCTU),
Institute of Applied Health Research, Public Health Building, University of Birmingham, Edgbaston, Birmingham, B15 2TT

For peer review only

UNIVERSITY OF BIRMINGHAM

The PIMMS-WL Trial

Feasibility and acceptability of a brief routine weight management intervention for postnatal women embedded within the national child immunisation programme in primary care: randomised controlled cluster feasibility trial with nested qualitative study.

Patient Consent Form A
Version 4.0 1st February 2018

PIMMS-WL Trial No: ☐☐☐ - ☐☐☐☐ Patient Initials: ☐☐

Please initial each box to indicate your consent

**1.**	I confirm that I have read and understood the information sheet for the **PIMMS-WL** trial (Version __, dated DD/MM/YYYY). I have had the opportunity to consider the information, ask questions, and have had these answered satisfactorily.	☐
**2.**	I understand that my participation is voluntary and that, if I take part, I may withdraw at any time, without giving any reason, and without the standard of my medical care or legal rights being affected. I understand that data collected up to my time of withdrawal may be used.	☐
**3.**	Data collected that identifies me and my newborn baby by name, including my contact details and a copy of my consent form, will be transferred from where it is collected and stored at the University of Birmingham Clinical Trials Unit (BCTU). I agree to the transfer and storage of this data for use in the **PIMMS-WL** trial.	☐
**4.**	I understand that the study researchers may contact me regarding the study.	☐
**5.**	I agree for the **PIMMS-WL** trial team to collect my newborn child's immunisation attendance data from my GP records for the purposes of this research study only.	☐
**6.**	I give permission for my data collected during the study to be looked at by responsible individuals from the **PIMMS-WL** trial office at the University of Birmingham Clinical Trials Unit (BCTU), from the sponsor, from regulatory authorities or from the NHS Trust, where it is relevant to my taking part in this research. I understand that all information collected will be used for medical research only and that I will not be identified in any way in the analysis and reporting of the results.	☐
**7.**	I agree to my GP being informed of my participation in the **PIMMS-WL** trial, a copy of my signed consent form to be sent, and my GP to be contacted to obtain any medical history relevant to this study.	☐
**8.**	I agree to take part in the **PIMMS-WL** trial.	☐

Name of Participant: ....

Signature: .... Date:...

Name of Person taking consent: ....

Signature: .... Date:...

Original to be filed in the Site File; 1 copy for patient; 1 copy to be kept with patient's GP record; 1 copy to be sent to BCTU
Please return a copy of this form to: The PIMMS-WL Trial, Birmingham Clinical Trials Unit (BCTU),
Institute of Applied Health Research, Public Health Building, University of Birmingham, Edgbaston, Birmingham, B15 2TT

For peer review only

UNIVERSITY OF BIRMINGHAM

The PIMMS-WL Trial

Feasibility and acceptability of a brief routine weight management intervention for postnatal women embedded within the national child immunisation programme in primary care: randomised controlled cluster feasibility trial with nested qualitative study.

Patient Consent Form B
Version 4.0 1st February 2018

For peer review only

PIMMS-WL Trial No:	☐☐-☐☐☐☐	Patient Initials:	☐☐

Please initial each box to indicate your consent

**1.**	I confirm that I have read and understood the information sheet for the **PIMMS-WL** trial (Version __, dated DD/MM/YYYY). I have had the opportunity to consider the information, ask questions, and have had these answered satisfactorily.	☐
**2.**	I understand that my participation is voluntary and that, if I take part, I may withdraw at any time, without giving any reason, and without the standard of my medical care or legal rights being affected. I understand that data collected up to my time of withdrawal may be used.	☐
**3.**	Data collected that identifies me and my newborn baby by name, including my contact details and a copy of my consent form, will be transferred from where it is collected and stored at the University of Birmingham Clinical Trials Unit (BCTU). I agree to the transfer and storage of this data for use in the **PIMMS-WL** trial.	☐
**4.**	I understand that I will be asked to complete data online using the POWeR website at the University of Southampton. I consent to the **PIMMS-WL** research team to access, transfer and store this electronic data.	☐
**5.**	I understand that the study researchers may contact me regarding the study.	☐
**6.**	I agree for the **PIMMS-WL** trial team to collect my newborn child's immunisation attendance data from my GP records for the purposes of this research study only.	☐
**7.**	I give permission for my data collected during the study to be looked at by responsible individuals from the **PIMMS-WL** trial office at the University of Birmingham Clinical Trials Unit (BCTU), University of Southampton, from the sponsor, from regulatory authorities or from the NHS Trust, where it is relevant to my taking part in this research. I understand that all information collected will be used for medical research only and that I will not be identified in any way in the analysis and reporting of the results.	☐
**8.**	I agree to my GP being informed of my participation in the **PIMMS-WL** trial, a copy of my signed consent form to be sent, and my GP to be contacted to obtain any medical history relevant to this study.	☐
**9.**	I agree to take part in the **PIMMS-WL** trial.	☐

**OPTIONAL CONSENT:**	**Please initial one box only**	**I agree**	**I do not agree**

**10.**	I am willing for the nurse to audio record the part of my immunisation visits when she discusses the study with me. The recording and transcription will be securely transferred and stored at the University of Birmingham Clinical Trials Unit. The recording will be transcribed by an external company and analysed for the purposes of this research only.		

Name of Participant: ....

Signature: .... Date:.......................................

Name of Person taking consent: ....

Signature: .... Date:.......................................

Original to be filed in the Site File; 1 copy for patient; 1 copy to be kept with patient's GP record; 1 copy to be sent to BCTU
Please return a copy of this form to: The PIMMS-WL Trial, Birmingham Clinical Trials Unit (BCTU),
Institute of Applied Health Research, Public Health Building, University of Birmingham, Edgbaston, Birmingham, B15 2TT

For peer review only

Table 1: Detailed study objectives

1. In women who have recently given birth, assess the feasibility of delivering an intervention to promote self-management of weight loss, by self-monitoring of weight and signposting to an online weight management programme by practice nurses as part of the UK child immunisation programme
2. Assess recruitment to ensure a full-scale phase III cluster trial is feasible
3. Determine the extent of participant burden in completing the trial questionnaires
4. Determine the potential risk for intervention contamination (whether women in the control group spontaneously access the online programme) to assess if the main trial sample size will need to be adjusted to account for this
5. Determine levels of adherence to the intervention
6. Collect data on immunisation uptake rates (to check there is no difference in rates in both groups, adjusted for the normal rate in the practice)
7. To provide estimates of the variability in the primary outcome (weight) to inform the sample size for the phase III trial
8. Using semi structured interviews explore practice nurses' views about delivering the intervention and explore any variation in intervention delivery to ascertain if any adjustments to nurse training are required
9. Based on feedback from participants through interviews explore the acceptability of the intervention
10. Assess the impact of the intervention on breast feeding rates and psychological health in both groups
11. Explore the acceptability/validity of the ICECAP (ICEpop CAPability measure for Adults) for the cost effectiveness analysis in the phase III trial

For peer review only

Table 2: Inclusion and exclusion criteria

**Inclusion criteria**	**Exclusion criteria**
Aged 18 years or more	Mothers whose babies have died or have been removed from their care at birth
Women who are at least four weeks postnatal and who have not yet attended the first child immunisation appointment	Women who indicate they are already actively involved in a weight loss programme or weight management trial to lose weight
Planning to have their child immunised within the national immunisation programme	Unwilling to give consent to notify their GP
BMI 25kg/m^2 or more at the time of recruitment at the baseline home visit	Women who have been diagnosed with a serious mental health difficulty requiring hospitalisation in the past two years or been diagnosed with anorexia and/or bulimia in the past two years
Patient able and willing to provide written informed consent	

For peer review only

Table 3: STOP-GO traffic light criteria

Green light	• Recruitment rate ≥80% of the target (n=80; i.e. recruit at least 64 women), ≥50% of the intervention group weighs themselves weekly ≥60% of the time and ≥60% of participants have registered with POWeR. If all three criteria are met we will proceed to application for the full trial with the protocol unchanged (unless there is a clear message from the interviews that would improve the protocol).
Amber light	• Recruitment rate of 50-79% of the target (n=80; i.e. recruit between 40 and 63 women), 40-49% of the intervention group weighs themselves weekly 40-59% of the time and 40-59% of the intervention group registered with POWeR. If one or more of our amber light criteria are met, we will plan to adapt the protocol in light of the results of the feedback from the interviews and our experience to improve whichever criteria are not at the "green-light" level before proceeding to a full trial. In discussion with the trial steering committee we will assess whether adaption of the protocol will require further assessment before progressing.
Red light	• Recruitment rate of <50% of the target (n=80; i.e. recruit less than 40 women), <40% of the intervention group weighs themselves weekly 40-59% of the time and <40% of the intervention group have registered with POWeR. If one or more of these criteria are met, we would consider the current protocol not feasible and not progress to a full RCT with the current design. An additional red light criteria would be concerns from the TSC that immunisation rates have been adversely affected.

For peer review only

Table 4: Qualitative study objectives

1.	To explore whether the child immunisation appointments are an appropriate setting for postnatal mother's weight to be monitored
2.	To capture mother's views on how useful the intervention was at helping them manage their weight
3.	To determine what elements of the intervention facilitated and/or impeded its acceptability
4.	Explore what participants found helpful and unhelpful
5.	To investigate what aspects of the intervention were acceptable and unacceptable to participants and practice nurses, as well as the reasons for these feelings and opinions
6.	To assess what components of the intervention may need to be amended, if any
7.	To assess if the intervention leads to the mothers experiencing any anxiety or psychological harm relating to their weight
8.	To capture nurse's views on the impact of delivering the intervention had on the structure and duration of the child immunisation appointment

For peer review only

BMJ Open

Protocol for the feasibility and acceptability of a brief routine weight management intervention for postnatal women embedded within the national child immunisation programme: randomised controlled cluster feasibility trial with nested qualitative study (PIMMS-WL)

Journal:	*BMJ Open*
Manuscript ID	bmjopen-2019-033027.R1
Article Type:	Protocol
Date Submitted by the Author:	16-Dec-2019

Complete List of Authors:	Parretti, Helen; University of Birmingham, Insititue of Applied Health Research<br>Ives, Natalie; University of Birmingham, BCTU<br>Tearne, Sarah ; University of Birmingham Clinical Trials Unit<br>Vince, Alexandra ; University of Birmingham Edgbaston Campus, Birmingham Clinical Trials Unit<br>Greenfield, Sheila; University of Birmingham Edgbaston Campus, Institute of Applied Health Research<br>Jolly, Kate; University of Birmingham, Institute of Applied Health Research<br>Jebb, Susan; University of Oxford, Nuffield Department of Primary Care Health Sciences<br>Frew, Emma; University of Birmingham, Health Economics<br>Yardley, Lucy; University of Southampton, Psychology; University of Bristol, School of Health Sciences<br>Little, Paul; University of Southampton, Primary Care and Population Science;<br>Pritchett, Ruth; University of Birmingham Institute of Applied Health Research<br>Daley, Amanda; Loughborough University, School of Sport, Exercise and Health Sciences

**Primary Subject Heading**:	Obstetrics and gynaecology
Secondary Subject Heading:	Nutrition and metabolism, Public health, General practice / Family practice
Keywords:	postnatal, weight, QUALITATIVE RESEARCH, randomised cluster trial, Obesity

I, the Submitting Author has the right to grant and does grant on behalf of all authors of the Work (as defined in the below author licence), an exclusive licence and/or a non-exclusive licence for contributions from authors who are: i) UK Crown employees; ii) where BMJ has agreed a CC-BY licence shall apply, and/or iii) in accordance with the terms applicable for US Federal Government officers or employees acting as part of their official duties; on a worldwide, perpetual, irrevocable, royalty-free basis to BMJ Publishing Group Ltd ("BMJ") its licensees and where the relevant Journal is co-owned by BMJ to the co-owners of the Journal, to publish the Work in this journal and any other BMJ products and to exploit all rights, as set out in our licence.

The Submitting Author accepts and understands that any supply made under these terms is made by BMJ to the Submitting Author unless you are acting as an employee on behalf of your employer or a postgraduate student of an affiliated institution which is paying any applicable article publishing charge ("APC") for Open Access articles. Where the Submitting Author wishes to make the Work available on an Open Access basis (and intends to pay the relevant APC), the terms of reuse of such Open Access shall be governed by a Creative Commons licence – details of these licences and which Creative Commons licence will apply to this Work are set out in our licence referred to above.

Other than as permitted in any relevant BMJ Author's Self Archiving Policies, I confirm this Work has not been accepted for publication elsewhere, is not being considered for publication elsewhere and does not duplicate material already published. I confirm all authors consent to publication of this Work and authorise the granting of this licence.

For peer review only

Protocol for the feasibility and acceptability of a brief routine weight management intervention for postnatal women embedded within the national child immunisation programme: randomised controlled cluster feasibility trial with nested qualitative study (PIMMS-WL)

Helen M Parretti[1], Natalie Ives[2], Sarah Tearne[2], Alexandra Vince[2], Sheila M Greenfield[1], Kate Jolly[1], Susan A Jebb[3], Emma Frew[1], Lucy Yardley[4,5], Paul Little[6], Ruth V Pritchett[1], Amanda J Daley[7]*

*Corresponding author

Professor Amanda Daley

School of Sport, Exercise and Health Sciences,

Loughborough University,

Loughborough,

LE11 3TU, UK

a.daley@lboro.ac.uk

1 Institute of Applied Health Research, University of Birmingham, Edgbaston, Birmingham, B15 2TT

2 Birmingham Clinical Trials Unit, Public Health Building, University of Birmingham, Edgbaston, Birmingham, B15 2TT

3 Nuffield Department of Primary Care Health Sciences, University of Oxford, Radcliffe Observatory Quarter, Woodstock Road, Oxford, OX2 6GG, UK and Oxford NIHR Biomedical Research Centre.

4 School of Psychological Science, University of Bristol, Priory Road, Clifton BS8 1TU, UK

5 Department of Psychology, Building 44 Highfield Campus, University of Southampton, SO17 1BJ, UK

6 Primary Care and Population Sciences, Aldermoor Health Centre, Aldermoor Close, Southampton, SO16 5ST

7 School of Sport, Exercise and Health Sciences, Loughborough University, Loughborough, LE11 3TU, UK

Main text word count: 4422

For peer review only - http://bmjopen.bmj.com/site/about/guidelines.xhtml

ABSTRACT

Introduction

On average women retain 5-9kg one year after giving birth which can increase the risk of later obesity and chronic diseases. Some previous trials in this population have been effective in reducing weight, but are too intensive and costly to deliver at scale. There is a need for low-cost interventions to facilitate weight loss in this population.

Methods and analysis

The primary aim is to assess the feasibility of delivering a weight management intervention for overweight/obese postnatal women within child immunisation appointments. We will conduct a randomised controlled cluster feasibility trial with a nested qualitative study to assess study recruitment and acceptability of the intervention. GP practice (cluster) will be the unit of randomisation, with practices randomised to offer usual care plus the intervention or usual care only. 80 women will be recruited.

The intervention group will be offered brief support that encourages self-management of weight when attending child immunisation appointments. Practice nurses will encourage women to weigh themselves weekly and record this, and to make healthy lifestyle choices through using an online weight management programme. Women will be advised to aim for 0.5-1kg/week weight loss. At each child immunisation the nurse will assess progress by weighing women. The comparator group will receive a healthy lifestyle leaflet.

Data on weight, body fat, depression, anxiety, body-image, eating behaviours and physical activity will be collected at baseline and follow-up. Women and nurses will be interviewed to ascertain their views about the intervention. The decision to proceed to the phase III trial will be based on pre-specified stop-go criteria.

Ethics and Dissemination

Data will be stored securely at the University of Birmingham. Results will be disseminated through academic publications and presentations and will inform a possible phase III trial. The National Research Ethics Committee approved the study protocol.

Trial Registration number: ISRCTN12209332

For peer review only - http://bmjopen.bmj.com/site/about/guidelines.xhtml

Strengths and limitations of this study:

- This is the first study to assess the feasibility of embedding a weight management intervention for postnatal women within usual care contacts in primary care.
- This study uses mixed methods and will draw on both quantitative and qualitative data to assess the feasibility and acceptability of the intervention.
- The intervention is based on self-regulation theory with external accountability.
- Rather than rely on self-reports, the study uses objective recordings from real time weighing scales to assess the frequency of self-weighing.

For peer review only

Key Words: postnatal, randomised cluster feasibility trial, obesity, weight, qualitative

For peer review only - http://bmjopen.bmj.com/site/about/guidelines.xhtml

INTRODUCTION

Pregnancy is associated with significant weight gain and many women report pregnancy as the critical point for the onset of excess weight. (1-2) The postnatal period is characterised not only by weight retention, but also susceptibility to further weight gain, which can significantly increase the risk of later obesity and serious chronic diseases.(3-5) There is also an association between postnatal weight retention and poor mental health.(6-8) It is estimated that on average women gain about 14-15kg during pregnancy and at 1 year after birth 5-9kg is retained.(2-3, 9-10) Of women who are overweight prior to conception, 44% are living with obesity by 1 year after giving birth, while 97% of women who are living with obesity prior to pregnancy remain so at 1 year postnatally.(5) Therefore, there is a need to intervene routinely in the postnatal period, to help women manage their weight after having a baby and minimise long-term health risks.

Several systematic reviews have identified the need for high quality trials on how to best help women to lose weight after having a baby.(11-16) Within these reviews, the vast majority of studies have recruited small sample sizes and evaluated intensive physical activity and/or dietary interventions delivered by specialists; while these types of interventions can be effective, they cannot be delivered to all the women who give birth each year in the UK, of whom nearly two-thirds will be living with overweight.(17) A recent systematic review of reviews of lifestyle interventions for postnatal weight management highlighted the lack of studies that have tested an intervention embedded within routine health care.(16)

Self-management strategies and weight loss

A systematic review of randomised controlled trials (RCTs) to determine the effectiveness of self-help interventions for weight loss (18), has shown that self-help interventions can lead to small, but significantly greater weight loss than unsupported attempts to lose weight at six months compared with minimal interventions. In the small group of studies providing data at 12 months, weight loss was no longer significant, suggesting that self-help interventions on their own may not be useful for sustaining long term weight loss, and that additional components, within weight loss interventions are required. Nonetheless, given the potential scalability and relatively low cost of this type of intervention, self-help programmes may be a useful component within a broader intervention to treat those who are overweight.

Self-monitoring of weight by regular weighing is common to many self-management interventions. The potential efficacy of regular weighing is based on the principles of self-regulation theory.(19-20) Self-regulation theory describes three distinct stages; self-monitoring; self-evaluation and self-reinforcement. Self-monitoring is a method of systematic self-observation, periodic measurement

w Only

and recording of target behaviours with the goal of increasing self-awareness. The awareness fostered during self-monitoring is an essential initial step in promoting and sustaining behaviour change. Strong evidence supports the role of self-monitoring as an effective strategy in the health behaviour change process. Reviews by Michie *et al.* of effective behavioural techniques for healthy eating, physical activity and reduction of alcohol consumption concluded that self-monitoring was effective alone, but when combined with other techniques the effect size nearly doubled.(21-22) In a systematic review of RCTs to examine the effectiveness of self-weighing as a strategy for weight loss, multi-component interventions including self-weighing compared to no/minimal control also resulted in mean differences of -3.7 kg (95% CI -4.6 to -2.9).(23)

Accountability/audit and feedback

There has been some evidence to suggest that adding accountability to a self-weighing programme increases weight loss.(23) In a review by Madigan *et al.*, in 13 of the included trials, the intervention group asked to weigh themselves knew that they were accountable to a therapist/researcher, while this was not the case in two trials. There was a significant difference in mean weight loss between intervention and control for those trials with accountability compared with those without (-4.0kg, 95% CI -5.0 to -3.0kg versus -2.3kg, 95% CI -3.1 to -1.5kg; p=0.007).(23) Participants in group weight loss programmes often report that it is the weekly weigh-in that is the most salient component of the programme, providing external accountability and keeping them committed to their diet and physical activity plan.(24) Based on this evidence, it can be hypothesised that including accountability/audit to self-help and self-monitoring interventions could further facilitate weight loss.

Internet-based weight management interventions

The widespread use of technology has led to an interest in digital health interventions, including for weight management. Systematic reviews of eHealth or web-based interventions for weight loss and/or maintenance in adults have found that these interventions can result in modest weight loss in individuals with overweight or obesity.(25-26) One such digital interventions is the POWeR online weight management programme.(27) The POWeR programme was designed for use with minimal additional support and was developed with extensive user feedback; the development process and content of the POWeR intervention is described elsewhere. (28-29) The POWeR programme has been shown to result in significant weight loss at 12 months in primary care patients with overweight when combined with brief nurse support.(27) It focuses principally on fostering users' self-regulation skills for autonomously self-managing their weight, rather than providing detailed dietetic advice. A recent systematic review concluded that internet-based weight management interventions for postnatal mothers appear to be beneficial, and survey study results have reported

For peer review only - http://bmjopen.bmj.com/site/about/guidelines.xhtml

that irrespective of age or socio-economic status, 90.5% of new mothers use the internet as a source of information with weight loss as the fourth most searched topic (20-31). Therefore, based on this evidence it may be hypothesised that including an online intervention may be beneficial for postnatal weight loss.

Study rationale

There is a need to support women to lose excess weight in the postnatal period. Given the large numbers of women who give birth each year either with pre-existing overweight or obesity, or who gain excess weight during this period, an intervention is needed that can be delivered at scale without significant implications for a healthcare workforce. In addition, the National Institute for Health and Care Excellence (NICE) has highlighted the low quality of previous research as a limitation to developing clinical guidance in this area.(32) Here we investigate the feasibility of a postnatal weight management intervention embedded within pre-existing healthcare contacts. This pragmatic intervention is based on existing evidence for the effectiveness of both self-monitoring and an online programme for weight management (POWeR).(19, 27)

Aims and objectives

The primary objective is to produce evidence that a large-scale phase III cluster RCT of a weight management intervention embedded in the national child immunisation programme (33) would be acceptable to women and feasible to conduct. Detailed objectives are listed in Supplementary Table 1. This protocol is reported according to the Standard Protocol Items Recommendations for Interventional Trials (SPIRIT) guidelines.(34)

METHODS AND ANALYSIS

Trial Design

PIMMS-WL is a feasibility cluster RCT to assess the feasibility and acceptability of a weight management intervention, based on self-regulation theory, in women who have recently given birth.(20) This will be assessed through a composite of quantitative and qualitative data. Key stages of the study are shown in Figure 1.

Trial Setting

This study will take place in Birmingham, UK between 01/09/2017 and 31/10/2019. The initial identification of women will take place within Birmingham Women and Children's NHS Foundation Trust and within participating GP practices, and the trial will take place within GP practices around the West Midlands. The baseline and follow-up assessments will be completed in participants' homes.

For peer review only - http://bmjopen.bmj.com/site/about/guidelines.xhtml

Study participants

Women will be eligible if they are aged ≥18 years; are at least four weeks postnatal; have a BMI ≥25kg/m^2 at the time of recruitment; are able to provide written informed consent and do not meet any of the exclusion criteria (Supplementary Table 2). Given that the intervention is embedded in immunisation appointments to be eligible women need to have not yet attended their child's first immunisation appointment and to be planning to have their child immunised.

Consent

Written informed consent into the PIMMS-WL trial will be a two stage process conducted by a researcher at the baseline home visit (see Appendix 1 for Informed Consent Forms). First, written informed consent will be sought for screening to confirm all eligibility criteria are met. Once all eligibility criteria have been confirmed, participants will be invited to give their written informed consent to be enrolled into the RCT.

Sample Size and Recruitment

This is a feasibility trial therefore a formal sample size calculation has not been conducted. The trial is not designed or powered to detect a statistically significant difference in effectiveness between the two treatment groups.

We will recruit 10-12 GP practices (clusters) across Birmingham, UK and the list of participating practices will be held by Birmingham Clinical Trials Unit (BCTU). We will then aim to recruit 80 participants from these GP practices over eight months. Potentially eligible participants will be identified initially from medical records at Birmingham Women's and Children's NHS Foundation Trust (BWCH). Initial screening of medical records by BWCH will confirm the women is aged ≥18 years, has given birth at least four weeks previously, and is registered at a participating GP practice. We will exclude mothers whose babies have died or have been removed from their care.

A trial invitation letter and participant information sheet (PIS) will be mailed to potentially eligible women by BWCH, which includes a telephone number to call or a reply slip to return if they are interested in participating. Interested women will undergo telephone screening for eligibility. For women who fulfil the initial screening criteria and are still interested in taking part in the trial, a baseline home visit with a researcher will be organised.

Some additional recruitment strategies may also be used if required:

- posters and flyers advertising the study will be displayed in waiting rooms at participating GP practices.

For peer review only - http://bmjopen.bmj.com/site/about/guidelines.xhtml

- participants may be informed about the trial directly at appointments with the GP or other health care professional post-delivery and prior to the 2-month immunisation appointment in participating GP practices
- a researcher may be available at the GP practice on specific clinic dates. The PIMMS-WL researcher will provide potential participants with the letter of invitation and PIS directly either to read at the GP practice or for the participant to take home.

If, after having read the letter of invitation and PIS, a woman is interested in participating, they will contact the PIMMS-WL research team for telephone screening (or be screened by the PIMMS-WL researcher at the GP practice) and a baseline home visit will be arranged as described above.

Randomisation

The unit of randomisation is the GP practice (cluster). GP practices will be randomised in a 1:1 ratio to provide the usual care plus a weight management intervention or usual care only (comparator group) using minimisation for GP practice list size (large: 6000 or more; small: under 6000 patients) and Index of Multiple Deprivation (IMD) rank score.(35) The IMD will be based on the postcode of the GP practice; the IMD rank score ranges from 1 to 32,844 and will be divided into tertiles of high, medium and low levels of deprivation. BCTU will create the randomisation list to allocate GP practices to the two trial groups.

Trial intervention (usual care plus weight management group)

The intervention will be delivered within the national child immunisation programme to minimise costs and additional burden on the NHS workforce and avoid the need for additional contacts with health professionals at this busy time in women's lives. In the first year, babies are routinely immunised at two, three, four and twelve months of age; we plan to embed the intervention within these pre-existing immunisation contacts. The intervention group will be offered brief support that encourages active self-management of weight in the postnatal period when they attend their practice to have their child immunised during the first year of life. The intervention involves motivation and support by nurses to encourage women in the postnatal period to make healthier lifestyle choices through self-monitoring of weight and signposting to an online weight management programme (POWeR).(27)

Women will be provided with weighing scales and asked to weigh themselves weekly. The intervention will be delivered until the third immunisation appointment. Women will be advised and encouraged to record their weight on a weight record card that will be attached to the child health record (the 'red book') and also within the POWeR online programme.(27). The 'red book' includes a record of infant examinations and investigations, growth and immunisation history.

For peer review only - http://bmjopen.bmj.com/site/about/guidelines.xhtml

Weight loss goals

No clinical guidelines exist for rates of healthy weight loss for postnatal women, but for the general population NICE recommend 0.5-1kg per week.(36) Women will be advised to aim for 0.5-1kg per week weight loss until they have achieved a BMI less than 25 kg/m^2 and are no heavier than their self-reported pre-pregnant weight.

Accountability

Practice nurses will weigh the women at each child immunisation visit and record this weight as a source of regular external accountability. They will not provide any weight management advice.

Online weight loss programme (POWeR: Positive Online Weight Reduction)

Nurses will signpost women to the POWeR online weight loss programme for support and assistance with goal setting, action planning and implementation of changes to their lifestyle. Information about breast feeding and weight loss (such as diet, physical activity and rate of recommended weight loss) will be added to the programme for the purpose of this trial. Women will be encouraged to use the website weekly to track their weight, set and review eating and physical activity goals, and receive personalised advice.

Training of practice nurses

All nurses who deliver child immunisations at intervention practices will be trained to deliver the intervention following a standard protocol. A training manual will be provided, with instructions for the tasks to be undertaken by nurses during immunisation appointments.

Intervention fidelity

Two immunisation/intervention consultations per nurse will be audio recorded so that intervention fidelity can be assessed against an intervention checklist criteria, and the length of time taken to deliver the intervention monitored.

Usual care group

Women enrolled into the trial and registered at GP practices in the usual care comparator group will receive brief written information about following a healthy lifestyle using the NHS EatWell guide leaflet.(37)

Masking

It is not possible to mask participants or those providing the intervention to group allocation. In this feasibility study, it is also not possible to mask the outcome assessor, as the same member of the PIMMS-WL research team will need to undertake both the baseline and follow-up home visit to collect data. We do not believe this will introduce bias, as the aim of this study is to assess the

For peer review only - http://bmjopen.bmj.com/site/about/guidelines.xhtml

feasibility of undertaking a large phase III cluster RCT, and these outcomes are not affected by knowledge of group allocation and the data relating to the feasibility outcomes are not collected during the home visits. The proposed primary outcome for the phase III trial is weight which is objectively measured using scales.

Baseline assessment and follow-up

Baseline visit

The baseline visit will occur between 6-7 weeks postnatally and before the first immunisation visit.

Follow-up visit

A follow-up visit will take place three months post baseline. Women in the intervention group will be invited to participate in the qualitative study interview (see below). A £20 gift voucher will be given to participants as reimbursement for any inconvenience and expenses incurred.

Patient and public Involvement

Members of the CLAHRC-WM maternity patient and public involvement (PPI) group (PRIME) provided input into the development of this study. They gave advice on the relevance of the study question based on their experiences and their assessment of the burden of the intervention on potential participants. Two members of the public will be invited to join the Trial Steering Committee (TSC). The PPI group will meet three times a year and will provide input into the topic guide for the qualitative interviews and have input into and review all patient facing documents. They will review all dissemination materials and be involved in the dissemination of the trial results.

Primary outcome

The primary outcome is the feasibility of undertaking a full-scale phase III cluster RCT, and will include assessment of the following:

- whether the trial is appealing to women (via assessment of the recruitment rate to ensure a full phase III trial is feasible);
- whether the intervention is acceptable;
- whether the intervention has any adverse impact on infant immunisation rates;
- the number of women who complete the trial and complete the trial questionnaires.

The decision to proceed will be based on pre-specified stop-go criteria (see Supplementary Table 3).

Outcome data

Outcome data will be collected at baseline and three months follow-up (except height (baseline only)). While this feasibility trial will not be powered to detect meaningful differences in outcome

For peer review only - http://bmjopen.bmj.com/site/about/guidelines.xhtml

measures, it will give us the opportunity to ensure that there are no issues with the completion of these measures in a main trial. Height and weight will be measured at baseline to calculate BMI. Weight and percentage body fat will be assessed using a Tanita SC-240MA analyser using a standardised procedure (e.g. light clothing). Demographic (e.g. age, ethnicity, socioeconomic status), lifestyle and pregnancy related information (e.g. history of pregnancy related problems, mode of delivery), infant feeding practices, and maternal sleeping patterns will be collected. Anxiety and depression will be assessed using the Hospital Anxiety and Depression Scale, body image using the Body Image State Scale, eating habits using the three-factor eating questionnaire and self-reported physical activity will be measured using the Pregnancy Physical Activity Questionnaire.(38-41)

At follow-up, self-weighing will be assessed using the Daily Self-Weighing Perceptions Questionnaire and weight control strategies using the Weight Control Strategies Scale.(42) We will also record whether the usual care group have accessed the POWeR website to assess any intervention contamination. To inform the design of the economic evaluation in the phase III trial, we will explore the acceptability of the ICECAP instrument (43), a broader measure of health than the EQ-5D (44) that focuses on general wellbeing. This will be done by assessing rates of completion of the ICECAP, and incorporating questions about the acceptability of the ICECAP into the qualitative interviews.

Eating behaviours (both groups)

Our hypothesis is that regular self-weighing and monitoring of weight leads to the development of self-regulation and conscious cognitive energy restraint.(20,45) Using the revised three-factor eating questionnaire (41), we will examine if feedback from self-weighing has led to development of conscious cognitive energy restraint of eating. We will also measure uncontrolled eating and emotional eating.

Weight control strategies (both groups)

Weight control strategies will be assessed at follow-up in both groups using the Weight Control Strategies Scale. As well as a total score, the domains of dietary choices, self-monitoring strategies, physical activity and psychological coping are measured.(42)

Objective assessment of self-weighing/adherence (intervention group)

As an objective measure of compliance, the intervention group will be given weighing scales (BodyTrace, USA) that objectively record weight every time participants weigh themselves and then this information is remotely transmitted back to the research team at the University of Birmingham. These scales are only included as an objective process measure of adherence to self-weighing and

For peer review only - http://bmjopen.bmj.com/site/about/guidelines.xhtml

we will not provide any feedback to participants, nor will we monitor changes in weight during the trial follow-up period.

Weight record cards (intervention group)

The intervention group will complete weight record cards and we will collect these from participants as a measure of intervention implementation at the follow-up visit. The record cards will also tell us how much of the intervention was delivered by practice nurses per protocol.

Perceptions of regular self-weighing (intervention group)

In the intervention group, we will use five items from Steinberg's Daily Self-Weighing Perceptions Questionnaire (46) to measure perceptions of regular monitoring of weight at follow-up.

Use of the POWeR online programme (intervention group)

Using participants' unique usernames POWeR automatically records participants' usage of the website (e.g. pages visited, when/for how long, goals and weights entered and dates).

Attendance at immunisation appointments (both groups)

To ensure the intervention does not have an adverse impact on women attending the immunisation appointments all practices will be asked to provide data on all immunisation appointments attended by both groups. Any missed appointments will be investigated and a reason allocated by the GP surgery. We will also collect patient reported attendance at the immunisation appointments at the follow-up visit.

Assessment of harms

We will not be collecting adverse events for this trial. Serious Adverse Events (SAEs) which are attributable to the trial intervention will be documented and reported from the date of consent into the PIMMS-WL study until 30 days after the three month follow-up visit. SAEs or deaths related to pre-existing medical conditions are not considered related to the trial intervention and are therefore excluded from notification as SAEs.

Withdrawal

Participants will be aware that they can freely withdraw from the trial (or part of) at any time. Details of withdrawal will be clearly documented. Different types of withdrawal are defined as:

- The participant would like to withdraw from trial treatment but is willing to be followed up in accordance with the schedule of assessments
- The participant would like to withdraw from trial treatment and is not willing to be followed up or for any further data to be collected

For peer review only - http://bmjopen.bmj.com/site/about/guidelines.xhtml

• The participant wishes to withdraw completely and is not willing to have any of their data, including that already collected, to be used in any future trial analysis.

The details of withdrawal (date, reason and type of withdrawal) should be clearly documented in the source data.

If a recruited GP practice withdraws from participation in the trial prior to randomisation, they may be replaced by another practice. If a practice withdraws after randomisation, consideration will be given to replacing with another practice.

Statistical analysis

A separate statistical analysis plan will provide a more comprehensive description of the planned analyses. A brief outline of the planned analyses in relation to the STOP-GO criteria is provided below. The data analysis for this feasibility trial will be descriptive and mainly focus on confidence interval estimation, with no hypothesis testing performed.

The recruitment rate will be presented as a percentage based on the number of participants who took part in the trial divided by the target recruitment (n=80). BWCH will also provide data on the number of invitation letters sent, along with data on the age and ethnicity (in summary format) of the women who were sent an invitation letter.

The quantitative assessment of whether the intervention is acceptable will be based on the adherence of women to weekly self-weighing and use of the online POWeR programme. In the first instance, the objective recording of weight on the scales will be used to assess the frequency of self-weighing/adherence.

In addition, to check that the intervention has no adverse impact on infant immunisation rates, the proportion of babies who attended all three immunisation appointments will be reported for each practice (both groups) and the rate compared with the normal immunisation rate for the practice. Reasons for missed appointments will be summarised descriptively. Recruitment, adherence and immunisation rates will be summarised as proportions with 95% confidence intervals.

Qualitative study

We will use semi-structured interviews after trial follow-up to explore the views of women about the intervention and experiences of the practice nurses delivering the intervention.(47) Topic guides will be informed by existing literature.

We will purposively sample up to 15 women (*e.g.* age, ethnicity, socioeconomic status and BMI category at enrolment) and 8-10 practice nurses and ask them about their experiences of the trial, to allow for theme saturation to be reached. (48-49) Interviews will be conducted using a topic guide

For peer review only - http://bmjopen.bmj.com/site/about/guidelines.xhtml

which will explore the women's experiences of and attitudes to trial participation, their understanding and rating of the importance of postnatal weight management, strategies used for weight management and their use of the online weight programme. For practice nurses, we will explore their experiences of delivering the intervention, barriers and facilitators, the content and phrasing of discussions with women about weight and views about the approaches that work best and least well. The objectives of the qualitative study are summarised in Supplementary Table 4.

It is anticipated that interviews will take place face-to-face, but it is possible for logistical reasons that some will need to take place by telephone. Interviews will be recorded and transcribed with the permission of participants and thematically analysed using the Framework Method.(50) Data management will be facilitated by the use of QSR NVivo 11.(51) A list of overall and individual themes for women and practice nurses will be compiled to allow for cross group/individual comparison. Data collection and analysis will be iterative. All digitally recorded data that is collected during interviews will be recorded on an encrypted audio recording device. The transcriptions will be anonymised.

Decision to progress to the phase III trial

For the phase III trial to take place there needs to be evidence from this feasibility trial of meeting pre-specified STOP-GO rules. The trial is too small to include meaningful and sensitive STOP-GO criteria regarding the impact of the intervention on immunisation rates. However, we will check that the intervention has not adversely affected usual immunisation rates at each GP practice (as described above). The criteria to proceed to the phase III trial will be based on three criteria: recruitment rate; adherence to weekly self-weighing and registration with the online weight loss programme (POWeR) using the traffic light system shown in Supplementary Table 3.

Data collection and management

All data will be entered onto the PIMMS-WL trial database. All trial paperwork will be stored in lockable filing cabinets in a secure, swipe access part of the University of Birmingham. Password protected electronic databases, on secure University of Birmingham servers for trial data will have access limited to BCTU members of staff working on the trial. Further trial information on data management procedures is available on request via BCTU including data collection forms.

ETHICS AND DISSEMINATION

Ethics

The study will be conducted according to the principles of the Declaration of Helsinki and in accordance with other relevant national guidelines, regulations, and acts. The trial has been granted

For peer review only - http://bmjopen.bmj.com/site/about/guidelines.xhtml

research ethics committee approval by the West Midlands and Black Country National Research Ethics Committee (17/WM/0399, Protocol V9.0, 16/7/19).

Monitoring and oversight

The trial will form part of a portfolio of studies hosted and managed by BCTU. The University of Birmingham holds the relevant insurance for this study and is the nominated sponsor for this study. As this is a feasibility trial, we do not propose to hold a Data Monitoring and Ethics Committee. A TSC will provide overall supervision and oversight of the trial and ensure it is conducted in accordance with the principles of good clinical practice and relevant regulations. The TSC agreed the trial protocol and will agree to any protocol amendments. Submission of amendments and communication to the relevant stakeholders will be coordinated by BCTU. The TSC will include members who are independent of the investigators, their employing organisations, funders and sponsors. The TSC will monitor trial progress and conduct and advise on scientific credibility. An explicit purpose of the TSC is to monitor the immunisation uptake rates at the GP practices.

Dissemination

A lay summary of the study is available on the National Institute for Health Research website (https://bepartofresearch.nihr.ac.uk/trial-details/trial-detail?trialId=17314&location=&distance=). Final results of this feasibility study will be made publicly available through publication in peer-reviewed journals and presented at relevant conferences.

DISCUSSION

The postnatal period is characterised not only by weight retention, but also by susceptibility to further weight gain. Most trials in this population include small sample sizes and/or evaluated intensive weight loss interventions. These types of interventions cannot be offered to all women who give birth in the UK every year. This study will generate evidence as to whether a full phase III trial to test the effectiveness of this novel postnatal weight management intervention embedded within existing primary care contacts is feasible. Currently there is no national guidance for health professionals regarding postnatal weight management and the full trial testing the effectiveness of this intervention would have the potential to influence policy and future national guidelines.

DECLARATIONS

Competing interests

The authors declare that they have no competing interests.

Funding

For peer review only - http://bmjopen.bmj.com/site/about/guidelines.xhtml

This work is supported by the National Institute of Health Research (NIHR) Health Technology Assessment programme grant number 15/184/14. HMP is funded by a National Institute for Health Research Academic Clinical Lectureship.

Authors' contributions

AJD developed the original idea for the study along with HMP, KJ, SJ and RP. LY and PL contributed to the protocol in relation to digital technology. HMP, AJD, ST and NI drafted the paper with input from all authors. EF was responsible for writing the health economics aspects of the protocol. SG was involved in developing the qualitative component of the study protocol. NI was responsible for the statistical methodology within the protocol. ST and AV further contributed to the development of the protocol after funding was awarded. All authors read, commented and approved the final manuscript.

Acknowledgements

The authors would like to thank the lay representatives who contributed to the trial steering committee and the lay representatives who contributed to the study design.

Disclaimer

This report is independent research commissioned by the National Institute for Health Research. The views expressed in this publication are those of the authors and not necessarily those of the NHS, the National Institute for Health Research or the Department of Health.

Ethics approval

This manuscript is based on Protocol V9.0 dated 16/7/19–submitted and approved by the West Midlands and Black Country NHS Research Ethics Committee (17/WM/0399, approval date 17/7/19).

For peer review only - http://bmjopen.bmj.com/site/about/guidelines.xhtml

For peer review only

REFERENCES

1. Gore SA et al. The role of postpartum weight retention in obesity among women: a review of the evidence. Ann Behav Med 2003;26:149.
2. Walker LO. Managing excessive weight gain during pregnancy and the postpartum period. J Obstet Gynecol Neonatal Nurs 2007;36:490.
3. Walker LO et al. Do low income women attain their pre-pregnant weight by the 6th week of postpartum? Ethn Dis 2004;14:119.
4. Schmitt NM et al. The association of pregnancy and the development of obesity - results of a systematic review and meta-analysis on the natural history of postpartum weight retention. Int J Obes 2007;31:1642.
5. Prospective Studies Collaboration et al. Body-mass index and cause-specific mortality in 900 000 adults: collaborative analyses of 57 prospective studies. Lancet 2009;373:1083.
6. Beck C. The effects of postpartum depression on maternal-infant interactions: A meta analysis. Nurs Res 1995;44:298.
7. Cooper PJ, et al. Course and recurrence of postnatal depression. Br J Psychiatry 1995;166: 191-5.
8. Murray L. The impact of postnatal depression on infant development. J Child Psychol Psychiatry 1992;33:543-561.
9. NHS Information Centre for Health and Social Care. Statistics on obesity, physical activity and diet: England, 2011. Available at: http://data.gov.uk/dataset/statistics-on-obesity-physical-activity-and-diet-england-2011.
10. Althuizen E, et al. Postpartum behaviour as predictor of weight change from before pregnancy to one year postpartum. BMC Public Health 2011; 11:165.
11. Hoedjes M et al. Effect of postpartum lifestyle interventions on weight loss, smoking cessation and prevention of smoking relapse: a systematic review. Obstet Gynecol Surv 2010;65:10.
12. Fowles ER et al. Postpartum health promotion interventions: a systematic review. Nurs Res 2012;61:296.
13. van der Pligt P et al. Systematic review of lifestyle interventions to limit postpartum weight retention: implications for future opportunities to prevent maternal overweight and obesity following childbirth. Obes Rev. 2013;14:792.
14. Nascimento SL et al. The effect of physical exercise strategies on weight loss in postpartum women: a systematic review and meta-analysis. Int J Obes 2014;38:626.
15. Amorim Adegboye AR et al. Diet or exercise, or both, for weight reduction in women after childbirth. Cochrane Database of Syst Rev 2013.

For peer review only - http://bmjopen.bmj.com/site/about/guidelines.xhtml

16. Ferguson JA et al. Behavioural weight management interventions for postnatal women: A systematic review of systematic reviews of randomized controlled trials. Obes Rev 2019: doi.org/10.1111/obr.12834.
17. Office for National Statistics. Annual Mid-year Population Estimates, 2011 and 2012. Available at: http://www.ons.gov.uk/ons/rel/pop-estimate/population-estimates-for-uk--england-and-wales--scotland-and-northern-ireland/mid-2011-and-mid-2012/stb---mid-2011---mid-2012-uk-population-estimates.html#tab-Key-Points.
18. Hartmann-Boyce J et al. Self-help interventions for weight loss in overweight and obese adults: systematic review and meta-analysis. Am J Public Health 2015;105:3.
19. Kanfer FH. Self-monitoring: methodological limitations and clinical applications. J Consult Clin Psychol 1970;35:148.
20. Cameron LD et al. The self-regulation of health and illness behavior. London: Routledge; 2003.
21. Michie S et al. Effective techniques in healthy eating and physical activity interventions: a meta-regression. Health Psychol 2009;28:690.
22. Michie S et al. Identification of behaviour change techniques to reduce excessive alcohol consumption. Addiction. 2012;107:1431.
23. Madigan C et al. Is self weighing an effective weight management tool: systematic review and meta analysis. I J Beh Nut Phys Act 2015 21;12:104.
24. Ahern AL et al. Participants' explanatory model of being overweight and their experiences of 2 weight loss interventions. Ann Fam Med 2013;11:251.
25. Hutchesson MJ et al. eHealth interventions for the prevention and treatment of overweight and obesity in adults: a systematic review with meta-analysis. Obes Rev. 2015;16(5):376.
26. Kozak AT et al. Technology-based interventions for weight management: current randomized controlled trial evidence and future directions. J Behav Med. 2017;40(1):99.
27. Little P et al. An internet-based intervention with brief nurse support to manage obesity in primary care (POWeR+): a pragmatic, parallel-group, randomised controlled trial. Lancet Diabetes Endocrinol 2016; 4(10): 821.
28. Bradbury K et al. Using mixed methods to develop and evaluate an online weight management intervention. Br J Health Psychol. 2015; 20(1):45.
29. Yardley L et al. Integrating user perspectives into the development of a web-based weight management intervention. Clin Obes. 2012; 2(5-6):132.
30. Slomian J et al. The internet as a source of information used by women after childbirth to meet their need for information: A web-based survey. Midwifery. 2017;48:46.

For peer review only - http://bmjopen.bmj.com/site/about/guidelines.xhtml

31. Sherifali D et al. The Effectiveness of eHealth Technologies on Weight Management in Pregnant and Postpartum Women: Systematic Review and Meta-Analysis. J Med Internet Res. 2017;19(10):e337
32. National Institute for Health and Care Excellence. Public Health Guidance 27: Weight management before, during and after pregnancy: costings report. Available at: http://www.nice.org.uk/guidance/ph27/resources/ph27-weight-management-before-during-and-after-pregnancy-costing-report2.
33. Public Health England. Vaccine coverage and cover. Available at: https://www.gov.uk/government/collections/vaccine-uptake.
34. Chan A-W et al. SPIRIT 2013 Statement: Defining standard protocol items for clinical trials. Ann Intern Med 2013;158:200-207.
35. McLennan D et al. The English Indices of Deprivation 2010. Department for Communitites and Local Government. London. Available at: https://www.gov.uk/government/uploads/system/uploads/attachment_data/file/6320/1870718.pdf.
36. National Institute for Health and Care Excellence. Clinical Guidance189: Obesity: identification, assessment and management. Available at: https://www.nice.org.uk/guidance/cg189.
37. Public Health England. Eatwell Guide. Available at: https://www.gov.uk/government/publications/the-eatwell-guide
38. Zigmond AS, Snaith RP. The Hospital Anxiety and Depression Scale. *Acta Psychiatr Scand.* 1983, 67(6):361-70.
39. Chasan-Taber L et al. Development and validation of a pregnancy physical activity questionnaire. Med Sci Sports Exerc 2004;36:1750.
40. Cash TF et al. Beyond body image as a trait: the development and validation of the body image states scale. Eat Disord 2002;10:103.
41. de Lauzon B et al. The Three Factor Eating Questionnaire-R18 is able to distinguish among different eating patterns in a general population. J Nutr 2004;134:2372.
42. Pinto AM et al. Development and validation of the weight control strategies scale. Obesity 2013;21:2429.
43. Al-Janabi H et al. Development of a self-report measure of capability wellbeing for adults: the ICECAP-A. Qual Life Res 2012;21:167.
44. EuroQol Group. EuroQol--a new facility for the measurement of health-related quality of life. Health Policy 1990;16:199.
45. Butryn ML et al. Consistent self monitoring of weight: a key component of successful weight loss maintenance. Obesity 2007:15;3091.

For peer review only - http://bmjopen.bmj.com/site/about/guidelines.xhtml

46. Steinberg DM et al. The efficacy of a daily self-weighing weight loss intervention using smart scales and email. Obesity 2013;21:1789.
47. Jamshed S Qualitative research method-interviewing and observation J Basic Clin Pharm 2014; v.5(4).
48. Lawrence A et al. Purposeful sampling for qualitative data collection and analysis in mixed method implementation research Adm Policy Ment Health. 2015; 42(5): 533.
49. Morse JM. Determining sample size*. Qual Health Res* 2000;10;1:3.
50. Gale NK et al. Using the framework method for the analysis of qualitative data in multi-disciplinary health research *BMC Med Res Methodol* 2013**13**:117
51. QSR International Pty Ltd. NVIVO Qualitative data analysis software V.11. 2012.

For peer review only

For peer review only - http://bmjopen.bmj.com/site/about/guidelines.xhtml

Figure and tables

Figure 1: Study flow diagram

Supplementary Table 1: Detailed study objectives

Supplementary Table 2: Inclusion and exclusion criteria

Supplementary Table 3: STOP-GO traffic light criteria

Supplementary Table 4: Qualitative study objectives

For peer review only

For peer review only - http://bmjopen.bmj.com/site/about/guidelines.xhtml

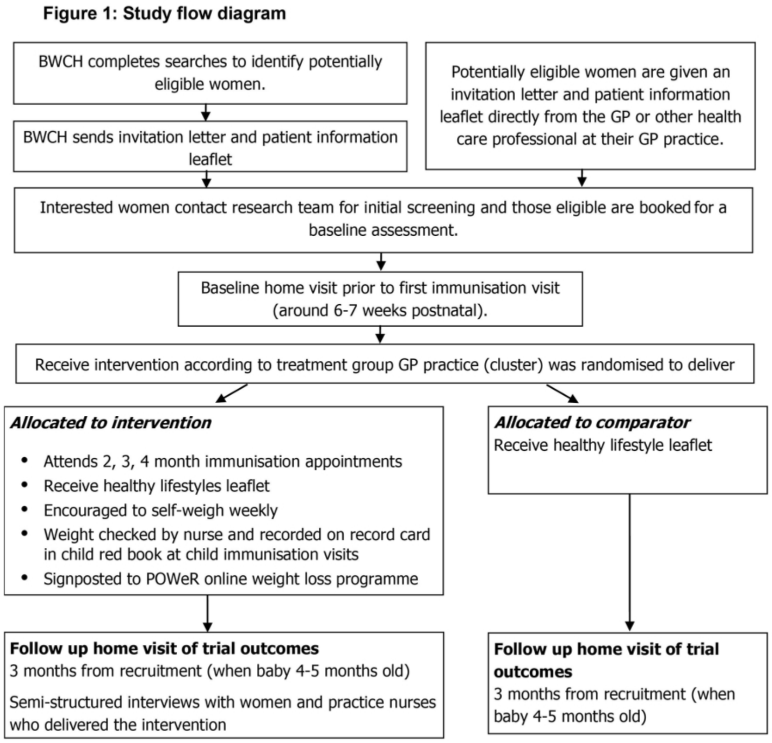

90x90mm (600 x 600 DPI)

Table 1: Detailed study objectives

1.	In women who have recently given birth, assess the feasibility of delivering an intervention to promote self-management of weight loss, by self-monitoring of weight and signposting to an online weight management programme by practice nurses as part of the UK child immunisation programme
2.	Assess recruitment to ensure a full-scale phase III cluster trial is feasible
3.	Determine the extent of participant burden in completing the trial questionnaires
4.	Determine the potential risk for intervention contamination (whether women in the control group spontaneously access the online programme) to assess if the main trial sample size will need to be adjusted to account for this
5.	Determine levels of adherence to the intervention
6.	Collect data on immunisation uptake rates (to check there is no difference in rates in both groups, adjusted for the normal rate in the practice)
7.	To provide estimates of the variability in the primary outcome (weight) to inform the sample size for the phase III trial
8.	Using semi structured interviews explore practice nurses' views about delivering the intervention and explore any variation in intervention delivery to ascertain if any adjustments to nurse training are required
9.	Based on feedback from participants through interviews explore the acceptability of the intervention
10.	Assess the impact of the intervention on breast feeding rates and psychological health in both groups
11.	Explore the acceptability/validity of the ICECAP (ICEpop CAPability measure for Adults) for the cost effectiveness analysis in the phase III trial

For peer review only

Table 2: Inclusion and exclusion criteria

**Inclusion criteria**	**Exclusion criteria**
Aged 18 years or more	Mothers whose babies have died or have been removed from their care at birth
Women who are at least four weeks postnatal and who have not yet attended the first child immunisation appointment	Women who indicate they are already actively involved in a weight loss programme or weight management trial to lose weight
Planning to have their child immunised within the national immunisation programme	Unwilling to give consent to notify their GP
BMI 25kg/m^2 or more at the time of recruitment at the baseline home visit	Women who have been diagnosed with a serious mental health difficulty requiring hospitalisation in the past two years or been diagnosed with anorexia and/or bulimia in the past two years
Patient able and willing to provide written informed consent	

For peer review only

Table 3: STOP-GO traffic light criteria

Green light	• Recruitment rate ≥80% of the target (n=80; i.e. recruit at least 64 women), ≥50% of the intervention group weighs themselves weekly ≥60% of the time and ≥60% of participants have registered with POWeR. If all three criteria are met we will proceed to application for the full trial with the protocol unchanged (unless there is a clear message from the interviews that would improve the protocol).
Amber light	• Recruitment rate of 50-79% of the target (n=80; i.e. recruit between 40 and 63 women), 40-49% of the intervention group weighs themselves weekly 40-59% of the time and 40-59% of the intervention group registered with POWeR. If one or more of our amber light criteria are met, we will plan to adapt the protocol in light of the results of the feedback from the interviews and our experience to improve whichever criteria are not at the "green-light" level before proceeding to a full trial. In discussion with the trial steering committee we will assess whether adaption of the protocol will require further assessment before progressing.
Red light	• Recruitment rate of <50% of the target (n=80; i.e. recruit less than 40 women), <40% of the intervention group weighs themselves weekly 40-59% of the time and <40% of the intervention group have registered with POWeR. If one or more of these criteria are met, we would consider the current protocol not feasible and not progress to a full RCT with the current design. An additional red light criteria would be concerns from the TSC that immunisation rates have been adversely affected.

For peer review only

Table 4: Qualitative study objectives

1.	To explore whether the child immunisation appointments are an appropriate setting for postnatal mother's weight to be monitored
2.	To capture mother's views on how useful the intervention was at helping them manage their weight
3.	To determine what elements of the intervention facilitated and/or impeded its acceptability
4.	Explore what participants found helpful and unhelpful
5.	To investigate what aspects of the intervention were acceptable and unacceptable to participants and practice nurses, as well as the reasons for these feelings and opinions
6.	To assess what components of the intervention may need to be amended, if any
7.	To assess if the intervention leads to the mothers experiencing any anxiety or psychological harm relating to their weight
8.	To capture nurse's views on the impact of delivering the intervention had on the structure and duration of the child immunisation appointment

For peer review only

Appendix: Informed Consent Forms

PIMMS-WL

UNIVERSITY OF BIRMINGHAM

The PIMMS-WL Trial

Feasibility and acceptability of a brief routine weight management intervention for postnatal women embedded within the national child immunisation programme in primary care: randomised controlled cluster feasibility trial with nested qualitative study.

Patient Screening Consent Form
Version 4.0 1st February 2018

PIMMS-WL Trial No: ☐☐☐ - ☐☐☐☐ Patient Initials: ☐☐

Please initial each box to indicate your consent

**1.**	I confirm that I have read and understood the information sheet for the **PIMMS-WL** trial (Version __, dated DD/MM/YYYY). I have had the opportunity to consider the information, ask questions, and have had these answered satisfactorily.	☐
**2.**	I understand that my participation is voluntary, that I am not obliged to take part in the full **PIMMS-WL** trial, and the standard of my medical care or legal rights will not be affected.	☐
**3.**	I understand that data collected that identifies me by name, including my contact details and my consent form, will be transferred from where it is collected and stored at the University of Birmingham Clinical Trials Unit (BCTU). I agree to the transfer and storage of this data for use in the **PIMMS-WL** trial.	☐
**4.**	I give permission for my data collected during the study to be looked at by responsible individuals from the **PIMMS-WL** trial office at the University of Birmingham Clinical Trials Unit (BCTU), from the sponsor, from regulatory authorities or from the NHS Trust, where it is relevant to my taking part in this research. I understand that all information collected will be used for this research only and that I will not be identified in any way in the analysis and reporting of the results.	☐
**5.**	I agree to take part in screening in order to determine my eligibility for participation for the **PIMMS-WL** study and I understand that I may not be suitable to take part as detailed in the information sheet.	☐

Name of Participant: ....

For peer review only

Signature: ………………………………………………………… Date:…………………………………

Name of Person taking consent: ………………………………………………………………………….

Signature: ………………………………………………………… Date:…………………………………

Original to be filed in the Investigator's Site File; 1 copy for patient;
1 copy to be kept with patient's GP record; 1 copy to be sent to BCTU
Please return a copy of this form to: The PIMMS-WL Trial, Birmingham Clinical Trials Unit (BCTU),
Institute of Applied Health Research, Public Health Building, University of Birmingham, Edgbaston, Birmingham, B15 2TT

For peer review only

UNIVERSITY OF BIRMINGHAM

PIMMS-WL

The PIMMS-WL Trial

Feasibility and acceptability of a brief routine weight management intervention for postnatal women embedded within the national child immunisation programme in primary care: randomised controlled cluster feasibility trial with nested qualitative study.

Patient Consent Form A

Version 4.0 1st February 2018

For peer review only

PIMMS-WL Trial No:			-					Patient Initials:		

Please initial each box to indicate your consent

**1.**	I confirm that I have read and understood the information sheet for the **PIMMS-WL** trial (Version ___, dated DD/MM/YYYY). I have had the opportunity to consider the information, ask questions, and have had these answered satisfactorily.	
**2.**	I understand that my participation is voluntary and that, if I take part, I may withdraw at any time, without giving any reason, and without the standard of my medical care or legal rights being affected. I understand that data collected up to my time of withdrawal may be used.	
**3.**	Data collected that identifies me and my newborn baby by name, including my contact details and a copy of my consent form, will be transferred from where it is collected and stored at the University of Birmingham Clinical Trials Unit (BCTU). I agree to the transfer and storage of this data for use in the **PIMMS-WL** trial.	
**4.**	I understand that the study researchers may contact me regarding the study.	
**5.**	I agree for the **PIMMS-WL** trial team to collect my newborn child's immunisation attendance data from my GP records for the purposes of this research study only.	
**6.**	I give permission for my data collected during the study to be looked at by responsible individuals from the **PIMMS-WL** trial office at the University of Birmingham Clinical Trials Unit (BCTU), from the sponsor, from regulatory authorities or from the NHS Trust, where it is relevant to my taking part in this research. I understand that all information collected will be used for medical research only and that I will not be identified in any way in the analysis and reporting of the results.	
**7.**	I agree to my GP being informed of my participation in the **PIMMS-WL** trial, a copy of my signed consent form to be sent, and my GP to be contacted to obtain any medical history relevant to this study.	
**8.**	I agree to take part in the **PIMMS-WL** trial.	

Name of Participant: ....

Signature: .... Date:..

Name of Person taking consent: ....

Signature: .... Date:..

Original to be filed in the Site File; 1 copy for patient; 1 copy to be kept with patient's GP record; 1 copy to be sent to BCTU

Please return a copy of this form to: The PIMMS-WL Trial, Birmingham Clinical Trials Unit (BCTU), Institute of Applied Health Research, Public Health Building, University of Birmingham, Edgbaston, Birmingham, B15 2TT

UNIVERSITY OF BIRMINGHAM

PIMMS-WL

The PIMMS-WL Trial

Feasibility and acceptability of a brief routine weight management intervention for postnatal women embedded within the national child immunisation programme in primary care: randomised controlled cluster feasibility trial with nested qualitative study.

Patient Consent Form B

Version 4.0 1st February 2018

**PIMMS-WL** Trial No:	_ _ _ - _ _ _ _	Patient Initials:	_ _

Please initial each box to indicate your consent

**1.**	I confirm that I have read and understood the information sheet for the **PIMMS-WL** trial (Version __, dated DD/MM/YYYY). I have had the opportunity to consider the information, ask questions, and have had these answered satisfactorily.	
**2.**	I understand that my participation is voluntary and that, if I take part, I may withdraw at any time, without giving any reason, and without the standard of my medical care or legal rights being affected. I understand that data collected up to my time of withdrawal may be used.	
**3.**	Data collected that identifies me and my newborn baby by name, including my contact details and a copy of my consent form, will be transferred from where it is collected and stored at the University of Birmingham Clinical Trials Unit (BCTU). I agree to the transfer and storage of this data for use in the **PIMMS-WL** trial.	
**4.**	I understand that I will be asked to complete data online using the POWeR website at the University of Southampton. I consent to the **PIMMS-WL** research team to access, transfer and store this electronic data.	
**5.**	I understand that the study researchers may contact me regarding the study.	
**6.**	I agree for the **PIMMS-WL** trial team to collect my newborn child's immunisation attendance data from my GP records for the purposes of this research study only.	
**7.**	I give permission for my data collected during the study to be looked at by responsible individuals from the **PIMMS-WL** trial office at the University of Birmingham Clinical Trials Unit (BCTU), University of Southampton, from the sponsor, from regulatory authorities or from the NHS Trust, where it is relevant to my taking part in this research. I understand that all information collected will be used for medical research only and that I will not be identified in any way in the analysis and reporting of the results.	
**8.**	I agree to my GP being informed of my participation in the **PIMMS-WL** trial, a copy of my signed consent form to be sent, and my GP to be contacted to obtain any medical history relevant to this study.	
**9.**	I agree to take part in the **PIMMS-WL** trial.	

**OPTIONAL CONSENT:**	**Please initial one box only**	**I agree**	**I do not agree**

For peer review only

**10.**	I am willing for the nurse to audio record the part of my immunisation visits when she discusses the study with me. The recording and transcription will be securely transferred and stored at the University of Birmingham Clinical Trials Unit. The recording will be transcribed by an external company and analysed for the purposes of this research only.		

Name of Participant: ....

Signature: .... Date:.......................................

Name of Person taking consent: ....

Signature: .... Date:.......................................

Original to be filed in the Site File; 1 copy for patient; 1 copy to be kept with patient's GP record; 1 copy to be sent to BCTU
Please return a copy of this form to: The PIMMS-WL Trial, Birmingham Clinical Trials Unit (BCTU),
Institute of Applied Health Research, Public Health Building, University of Birmingham, Edgbaston, Birmingham, B15 2TT

For peer review only

STANDARD PROTOCOL ITEMS: RECOMMENDATIONS FOR INTERVENTIONAL TRIALS

SPIRIT 2013 Checklist: Recommended items to address in a clinical trial protocol and related documents*

Section/item	Item No	Description	Addressed on page number
**Administrative information**			
Title	1	Descriptive title identifying the study design, population, interventions, and, if applicable, trial acronym	________1______
Trial registration	2a	Trial identifier and registry name. If not yet registered, name of intended registry	_________2_____
	2b	All items from the World Health Organization Trial Registration Data Set	________N/A_____
Protocol version	3	Date and version identifier	_______15 and 16_
Funding	4	Sources and types of financial, material, and other support	_______15-16____
Roles and responsibilities	5a	Names, affiliations, and roles of protocol contributors	__1,16_____
	5b	Name and contact information for the trial sponsor	___15_________
	5c	Role of study sponsor and funders, if any, in study design; collection, management, analysis, and interpretation of data; writing of the report; and the decision to submit the report for publication, including whether they will have ultimate authority over any of these activities	____15-16______
	5d	Composition, roles, and responsibilities of the coordinating centre, steering committee, endpoint adjudication committee, data management team, and other individuals or groups overseeing the trial, if applicable (see Item 21a for data monitoring committee)	___15_____

For peer review only

**Introduction**			
Background and rationale	6a	Description of research question and justification for undertaking the trial, including summary of relevant studies (published and unpublished) examining benefits and harms for each intervention	____4-6_____
	6b	Explanation for choice of comparators	________9_____
Objectives	7	Specific objectives or hypotheses	_6, Tables 1 and 4
Trial design	8	Description of trial design including type of trial (eg, parallel group, crossover, factorial, single group), allocation ratio, and framework (eg, superiority, equivalence, noninferiority, exploratory)	__6_____
**Methods: Participants, interventions, and outcomes**			
Study setting	9	Description of study settings (eg, community clinic, academic hospital) and list of countries where data will be collected. Reference to where list of study sites can be obtained	___6, 7___
Eligibility criteria	10	Inclusion and exclusion criteria for participants. If applicable, eligibility criteria for study centres and individuals who will perform the interventions (eg, surgeons, psychotherapists)	_7 and Table 2___
Interventions	11a	Interventions for each group with sufficient detail to allow replication, including how and when they will be administered	__8-9____
	11b	Criteria for discontinuing or modifying allocated interventions for a given trial participant (eg, drug dose change in response to harms, participant request, or improving/worsening disease)	___12-13______
	11c	Strategies to improve adherence to intervention protocols, and any procedures for monitoring adherence (eg, drug tablet return, laboratory tests)	____10_____
	11d	Relevant concomitant care and interventions that are permitted or prohibited during the trial	_Table 2___
Outcomes	12	Primary, secondary, and other outcomes, including the specific measurement variable (eg, systolic blood pressure), analysis metric (eg, change from baseline, final value, time to event), method of aggregation (eg, median, proportion), and time point for each outcome. Explanation of the clinical relevance of chosen efficacy and harm outcomes is strongly recommended	10-14 and Table 3
Participant timeline	13	Time schedule of enrolment, interventions (including any run-ins and washouts), assessments, and visits for participants. A schematic diagram is highly recommended (see Figure)	7,10 and Figure 1

For peer review only - http://bmjopen.bmj.com/site/about/guidelines.xhtml

Sample size	14	Estimated number of participants needed to achieve study objectives and how it was determined, including clinical and statistical assumptions supporting any sample size calculations	____7_______
Recruitment	15	Strategies for achieving adequate participant enrolment to reach target sample size	____7-8_____

Methods: Assignment of interventions (for controlled trials)

Allocation:

Sequence generation	16a	Method of generating the allocation sequence (eg, computer-generated random numbers), and list of any factors for stratification. To reduce predictability of a random sequence, details of any planned restriction (eg, blocking) should be provided in a separate document that is unavailable to those who enrol participants or assign interventions	____8______
Allocation concealment mechanism	16b	Mechanism of implementing the allocation sequence (eg, central telephone; sequentially numbered, opaque, sealed envelopes), describing any steps to conceal the sequence until interventions are assigned	___8______
Implementation	16c	Who will generate the allocation sequence, who will enrol participants, and who will assign participants to interventions	__8 and Figure 1_
Blinding (masking)	17a	Who will be blinded after assignment to interventions (eg, trial participants, care providers, outcome assessors, data analysts), and how	___9-10______
	17b	If blinded, circumstances under which unblinding is permissible, and procedure for revealing a participant's allocated intervention during the trial	____N/A_____

Methods: Data collection, management, and analysis

Data collection methods	18a	Plans for assessment and collection of outcome, baseline, and other trial data, including any related processes to promote data quality (eg, duplicate measurements, training of assessors) and a description of study instruments (eg, questionnaires, laboratory tests) along with their reliability and validity, if known. Reference to where data collection forms can be found, if not in the protocol	___10-14___
	18b	Plans to promote participant retention and complete follow-up, including list of any outcome data to be collected for participants who discontinue or deviate from intervention protocols	___10, 12-13____

Data management	19	Plans for data entry, coding, security, and storage, including any related processes to promote data quality (eg, double data entry; range checks for data values). Reference to where details of data management procedures can be found, if not in the protocol	____14_____
Statistical methods	20a	Statistical methods for analysing primary and secondary outcomes. Reference to where other details of the statistical analysis plan can be found, if not in the protocol	___13-14____
	20b	Methods for any additional analyses (eg, subgroup and adjusted analyses)	____14_____
	20c	Definition of analysis population relating to protocol non-adherence (eg, as randomised analysis), and any statistical methods to handle missing data (eg, multiple imputation)	____N/A____
**Methods: Monitoring**			
Data monitoring	21a	Composition of data monitoring committee (DMC); summary of its role and reporting structure; statement of whether it is independent from the sponsor and competing interests; and reference to where further details about its charter can be found, if not in the protocol. Alternatively, an explanation of why a DMC is not needed	___15___
	21b	Description of any interim analyses and stopping guidelines, including who will have access to these interim results and make the final decision to terminate the trial	____N/A__
Harms	22	Plans for collecting, assessing, reporting, and managing solicited and spontaneously reported adverse events and other unintended effects of trial interventions or trial conduct	____12_____
Auditing	23	Frequency and procedures for auditing trial conduct, if any, and whether the process will be independent from investigators and the sponsor	____N/A__
**Ethics and dissemination**			
Research ethics approval	24	Plans for seeking research ethics committee/institutional review board (REC/IRB) approval	___14-15____
Protocol amendments	25	Plans for communicating important protocol modifications (eg, changes to eligibility criteria, outcomes, analyses) to relevant parties (eg, investigators, REC/IRBs, trial participants, trial registries, journals, regulators)	______15_______

For peer review only - http://bmjopen.bmj.com/site/about/guidelines.xhtml

Consent or assent	26a	Who will obtain informed consent or assent from potential trial participants or authorised surrogates, and how (see Item 32)	____7______
	26b	Additional consent provisions for collection and use of participant data and biological specimens in ancillary studies, if applicable	___N/A______
Confidentiality	27	How personal information about potential and enrolled participants will be collected, shared, and maintained in order to protect confidentiality before, during, and after the trial	_____14____
Declaration of interests	28	Financial and other competing interests for principal investigators for the overall trial and each study site	____15_____
Access to data	29	Statement of who will have access to the final trial dataset, and disclosure of contractual agreements that limit such access for investigators	___14_____
Ancillary and post-trial care	30	Provisions, if any, for ancillary and post-trial care, and for compensation to those who suffer harm from trial participation	___N/A_____
Dissemination policy	31a	Plans for investigators and sponsor to communicate trial results to participants, healthcare professionals, the public, and other relevant groups (eg, via publication, reporting in results databases, or other data sharing arrangements), including any publication restrictions	___15_______
	31b	Authorship eligibility guidelines and any intended use of professional writers	___16_____
	31c	Plans, if any, for granting public access to the full protocol, participant-level dataset, and statistical code	__N/A_______
**Appendices**			
Informed consent materials	32	Model consent form and other related documentation given to participants and authorised surrogates	__Appendix___
Biological specimens	33	Plans for collection, laboratory evaluation, and storage of biological specimens for genetic or molecular analysis in the current trial and for future use in ancillary studies, if applicable	___N/A_____

*It is strongly recommended that this checklist be read in conjunction with the SPIRIT 2013 Explanation & Elaboration for important clarification on the items. Amendments to the protocol should be tracked and dated. The SPIRIT checklist is copyrighted by the SPIRIT Group under the Creative Commons "Attribution-NonCommercial-NoDerivs 3.0 Unported" license.

For peer review only - http://bmjopen.bmj.com/site/about/guidelines.xhtml
